# *Additional sex combs* interacts with enhancer of zeste and trithorax and modulates levels of trimethylation on histone H3K4 and H3K27 during transcription of *hsp70*

**DOI:** 10.1186/s13072-017-0151-3

**Published:** 2017-09-19

**Authors:** Taosui Li, Jacob W. Hodgson, Svetlana Petruk, Alexander Mazo, Hugh W. Brock

**Affiliations:** 1 Department of Zoology, Life Sciences Institute, University of British Columbia, 2350 Health Science Mall, Vancouver, BC V6T 1Z4 Canada; 2 Department of Biochemistry and Molecular Biology, Thomas Jefferson University, Philadelphia, PA 19107 USA

**Keywords:** *Additional sex combs*, SET domain, Trithorax, Enhancer of zeste, *hsp70* transcriptional elongation, Histone trimethylation

## Abstract

**Background:**

Maintenance of cell fate determination requires the Polycomb group for repression; the trithorax group for gene activation; and the enhancer of trithorax and Polycomb (ETP) group for both repression and activation. *Additional sex comb*s (*Asx*) is a genetically identified ETP for the *Hox* loci, but the molecular basis of its dual function is unclear.

**Results:**

We show that in vitro, Asx binds directly to the SET domains of the histone methyltransferases (HMT) enhancer of zeste [E(z)] (H3K27me3) and Trx (H3K4me3) through a bipartite interaction site separated by 846 amino acid residues. In *Drosophila* S2 cell nuclei, Asx interacts with E(z) and Trx in vivo. *Drosophila Asx* is required for repression of heat-shock gene *hsp70* and is recruited downstream of the *hsp70* promoter. Changes in the levels of H3K4me3 and H3K27me3 downstream of the *hsp70* promoter in *Asx* mutants relative to wild type show that *Asx* regulates H3K4 and H3K27 trimethylation.

**Conclusions:**

We propose that during transcription Asx modulates the ratio of H3K4me3 to H3K27me3 by selectively recruiting the antagonistic HMTs, E(z) and Trx or other nucleosome-modifying enzymes to *hsp70*.

**Electronic supplementary material:**

The online version of this article (doi:10.1186/s13072-017-0151-3) contains supplementary material, which is available to authorized users.

## Background

Polycomb group (PcG) and trithorax group (trxG) proteins maintain gene repression and activation, respectively, during metazoan development [1–3]. In *Drosophila melanogaster*, *Asx* was originally identified as a PcG mutant because of prominent posterior transformations caused by derepression of *Hox* genes [4–6]. Subsequently, it was observed that embryos mutant for *Asx* exhibit both anterior and posterior transformations, because *Hox* genes are improperly activated and derepressed, respectively [6–8]. Consistent with this model, *Asx* mutants enhance the homeotic transformation of trxG [8] and PcG [9, 10] mutations. Genes with these characteristics have been termed enhancers of trithorax and Polycomb (ETP) [11, 12]. Genetic analysis suggests that Asx is required for both trxG and PcG function.

Various enzymatic activities are associated with trxG and PcG proteins, including trimethylation of histone H3 lysine 4 (H3K4) and H3K27 [13, 14]. Thus, one model to explain the ETP function of Asx is that it interacts directly with E(z) and Trx to regulate H3K4 and H3K27 methylation. An alternative model is that Asx affects trimethylation of H3K4 and H3K27 indirectly by regulating histone demethylases or acetyltransferases. In either model, Asx should be required to regulate levels of H3K4 and H3K27 methylation in vivo. To our knowledge, neither of these models has been tested on *Asx* or its mammalian homologs, perhaps because of difficulty of identifying a single locus at which both PcG and trxG proteins act at the same time in the same cell.

The *hsp70* gene is well characterized. Before heat-shock induction, the *hsp70* promoter region is maintained in a nucleosome-free conformation by the GAGA factor [15], with a paused Pol II located approximately 25 nucleotides downstream of the transcription starting site [16, 17]. The paused Pol II is phosphorylated at serine 5 (Ser-5) but not Ser-2 of the C-terminal domain (CTD) [18], showing that transcriptional elongation has not begun. In Drosophila, these events occur 2–4 h after egg deposition. Heat stress leads to recruitment of heat-shock factor (HSF) [19], positive transcription elongation factor b (P-TEFb), mediator and various elongation factors including Spt5, Spt6 and facilitates chromatin transcription (FACT) complex that contains Spt16 for synthesis of full-length transcripts [20–22]. P-TEFb contains Cdk9 that is required for Pol II CTD Ser-2 phosphorylation and transcription elongation [18].

Any temporal analysis of the heat-shock response in *Drosophila* later in development than the first 4 h of embryogenesis will have three phases: (1) an early phase corresponding to the switch from a promoter-paused state to elongation; (2) an intermediate phase that combines transcriptional initiation, promoter clearance and elongation; and (3) a phase in which transcription of heat-shock genes is terminated. Recruitment of the trithorax (Trx) protein complex, TAC1, is required to maintain high levels of transcriptional elongation and of H3K4 trimethylation at the *hsp70* promoter region [23]. The PcG gene *pleiohomeotic* (*pho*) is required to repress *hsp70* transcription after heat shock during termination phase [24]. Maternal deposition of *Asx* mRNA or Asx protein prevents analysis of transcriptional initiation or promoter clearance at *hsp70* in *Asx* mutants in early embryos (first 4 h of embryogenesis). In later embryos, when initiation, clearance and elongation are occurring simultaneously, it is difficult to distinguish these phases of transcription in chromatin immunoprecipitation experiments.

Here, we show that Asx interacts directly in vitro and associates in vivo with E(z) and Trx, suggesting a recruitment mechanism for modulation of trimethylation of H3K4 and H3K27 at *hsp70.* We also show that *hsp70* is an excellent target to investigate the molecular basis of Asx function as an ETP after 10 min of heat-shock induction. We show that at the *hsp70* locus, *Asx* represses *hsp70* transcription because *Asx* mutants induce induction of the heat-shock response, but unlike the PcG gene *pho*, it is not required in the termination phase. Asx is recruited downstream of the promoter following heat stress induction, but during the first 10 min of heat-shock induction, *Asx* repression of *hsp70* is independent of changes in levels of H3K4me3 and H3K27me3. Subsequently, *Asx* modulates levels of H3K4me3 and H3K27me3, notably at transition from elongation to termination during the heat-shock cycle. This, however, does not exclude the modulation of trimethylation by recruitment of other histone methyltransferases.

## Methods

### Fly culture, transgenic lines, embryo imaging and cell culture

Flies were maintained at 22 °C on standard cornmeal-sucrose medium containing Tegosept as a mold inhibitor. The *Asx* ^*3*^ allele was maintained over a *CyO twist*-*GAL4*, *UAS*-*eGFP* balancer chromosome. The *Asx* ^*3*^ mutant is a null mutation with 1.3-kb deletion in the middle of coding region that produces a truncated protein product of approximately 800 N-terminal amino acids [4, 25] (Hodgson, unpublished).

### Antibodies

The following antibodies were employed: sheep polyclonal anti-Asx (aa 75–95) IgG [25]; rabbit polyclonal anti-trimethyl histone H3K4 antibody (Active Motif, Cat.# 39159; 1:1000 dilution); rabbit polyclonal anti-trimethyl histone H3K27 (Millipore, Cat.# 07-449; 1:100 dilution); rabbit polyclonal IgG antibody (Abcam, Cat.# ab27478; 1:200 dilution) used as a negative control; rabbit polyclonal anti-E(z) (Santa Cruz, Cat.# sc-98265); rat polyclonal anti-Trx antisera and purified rabbit anti-Trx IgG (Mazo Lab). Rabbit anti-Asx antibody was raised against *Drosophila* Asx (aa 200–356) (Additional file 1: Text S1). The specificity of rabbit anti-Asx antibody was tested (Additional file 2: Fig. S1).

### Construction of Asx full length and deletion mutants for cell-free expression

The DNA fragment corresponding to the full-length Asx (1669 amino acid residues) was subcloned from pBS(KS+)–Asx(1–1669) as an *Nde*I–*Kpn*I fragment into the *Nde*I–*Sma*I sites of the vector pTβSTOP (kindly provided by Robert Tjian) downstream of the T7 promoter and β globin leader sequence to generate pTβSTOP-A(1–1669). An Asx COOH terminal deletion mutant A(1–1200) was generated by replacing the wild-type 1.59-kb *SpH*I–*Kpn*I sequence in pBS(KS+)–Asx with a 0.495-kb PCR fragment produced using the following primer pairs: forward: 5′-ccggattccttgg GCA AGA CAT TAC CAG TGG CT-3′ and reverse: 5′-ccggagtggtacc TCA CAT ATT ACT GTT GTG-3′. The pBS(KS+)–Asx(1–1200) was subsequently digested with *Kpn*I, end-repaired with T4 DNA polymerase and digested with *Nde*I. The truncated 3.6-kb Asx fragment was subcloned into the *Nde*I–*Sma*I site of pTβSTOP to generate pTβSTOP-A(1–1200). Four additional COOH terminal deletion fragments as well as four NH2 terminal deletion fragments of Asx were amplified from the pTβSTOP-A(1–1669) by PCR (Additional file 3: Table S1), subcloned into the *Nde*I–*Sma*I/*Eco*RV site of pTβSTOP and transformed into DH5α cells (Thermo Fisher). All plasmid constructs were expressed in the TNT-coupled T7 transcription/translation system (Promega) using rabbit reticulocyte lysate (IVT–RRL) for GST pull-down assays.

### Construction of GST fusions of AsxETSI-2 and the SET domains of E(z) and Trx

The SET domain of E(z) (aa residues 626–740) was amplified by PCR using the primer pair shown in Additional file 3: Table S1 and subcloned into the *Eco*RI–*Xho*I sites of pGEX-6P1 to generate pGEX-6P1-E(z)SET. The SET domain of Trx (aa 3608-3759) (kindly provided by Michael Kyba) was subcloned into the *Eco*RI–*Xho*I sites of pGEX-6P1 to generate pGEX-6P1-TrxSET. The E(z)/Trx SET domain interaction site 2 of Asx (AsxETSI-2), residues 1200–150 l, was amplified using primer pair in Additional file 3: Table S1 and subcloned into the *Eco*RI–*Xho*I sites of pGEX-6P-1 to generate pGEX-6P1-AsxETSI-2. The three GST fusion constructs were each transformed into the *E. coli* Rosetta 2(DE3) strain (Novagen) for expression.

### Expression and purification of GST-TrxSET and GST-AsxETSI-2 fusion proteins

Overnight cultures of 10 ml of cells transformed with either pGEX-6P-1, pGEX-6P1-TrxSET or pGEX-6P1-AsxETSI-2 were diluted into 240 ml Luria–Bertani (LB)/100 μg ml^−1^ Amp media and induced at an A_600_ of 0.8 units with 1 mM isopropyl-*β*-D-thio-galactoside (IPTG)/100 μM ZnSO_4_ for 14 h at 23 °C. The cells were centrifuged, washed with PBS, lysed in 20 ml buffer TEEZMG—0.5 M KCl, pH 7.9, supplemented with protease inhibitors and treated with 4 mg/ml lysozyme (Sigma) for 30 min at 4 °C. Each lysate was sonicated, diluted twofold with buffer TEEZMG—0.5 M KCl/protease inhibitors and centrifuged at 14,000 rpm for 20 min at 4 °C. Extracts of GST, GST-TrxSET or GST-AsxETSI-2 were recovered in the supernatant as soluble fractions named S1.

Twenty ml of each S1 fraction was rotated with 0.5 ml of GSH-agarose pre-equilibrated with buffer TEMZG—0.3 M KCl, pH 7.9, for 3 h at 4 °C. The protein-bound resin was washed three times with buffer PBSMG—0.3 M NaCl, pH 7.2, three times with buffer PBSMG—0.8 M NaCl, pH 7.2, three times with buffer TEMZG—0.5 M KCl, pH 7.9, and once with buffer TEMZ—0.1 M KCl, pH 9.0, at 4 °C. Proteins were eluted from a column with 6 ml of buffer Elut-TEMZ—0.1 M KCl, pH 9.0/10 mM reduced glutathione–NaOH and collected in 0.5 ml fractions. Peak fractions were pooled and dialyzed into buffer Dyl-TEEMZG—0.1 M KCl, pH 7.9, for 18 h at 4 °C and stored at −80 °C. Buffers used in these experiments are described in Additional file 4: Text S2.

### Expression and purification of GST-E(z)SET fusion protein

A 10-ml overnight culture of pGEX-6P1-E(z)SET was diluted into 240 ml LB/100 μg/ml Amp media and induced at an A_600_ of 0.8 units with 1 mM IPTG/100 μM ZnSO_4_ for 3 h at 23 °C. The cells were washed in PBS, lysed in 20 ml buffer TEEZG—0.5 M KCl, pH 7.9, supplemented with protease inhibitors and treated with 4 mg/ml lysozyme (Sigma) for 30 min at 4 °C. The lysate was sonicated, diluted twofold with buffer TEEZG—0.5 M KCl, pH 7.9/protease inhibitors and centrifuged at 14,000 rpm for 20 min at 4 °C.

The pellet was resuspended in 15 ml buffer TZS—0.3 M NaCl, pH 7.9 using a Dounce homogenizer and mixed on a nutator for 2 h at 4 °C to solubilize GST-E(z)SET. The homogenate was centrifuged at 14,000 rpm for 20 min at 4 °C, and the supernatant (PI-Ext) was diluted fivefold with buffer TZD—0.3 M NaCl, pH 7.9, to reduce the sarkosyl concentration to 2% in the presence of Triton X-100 and CHAPS [26]. Fifty ml of diluted P1-Ext was mixed with 0.5 ml of GSH-agarose and pre-equilibrated with buffer TZXSC—0.3 M NaCl, pH 7.9, for 3 h at 4 °C on a rotator. The protein-bound resin was washed three times with buffer TZGXSC—0.3 M KCl, pH 7.9, three times with buffer TZGXSC—0.6 M KCl, pH 7.9, and two times with buffer TZXSGC—0.1 M NaCl, pH 9.0, at 4 °C. Proteins were eluted from a column with 6 ml of buffer Elut-TZGXSC—0.1 M NaCl, pH 9.0/10 mM reduced glutathione–NaOH, pH 9.0, and collected in 0.5 ml fractions. Peak fractions were pooled and dialyzed into 1 l buffer Dyl-TZXS—0.1 M NaCl/15% glycerol for 18 h at 4 °C and stored at −80 °C.

### GST pull-down assay of ^35^S-Asx interaction with SET domains of Trx and E(z)

For each reaction, 60 μl of packed GSH-agarose equilibrated with immobilization buffer IM-A pH 7.9 was resuspended in 400 μl buffer IM-A and mixed with 6 μg of purified GST for 120 min at 4 °C on a nutator. The resin was pelleted at 6000 rpm for 2 min, washed two times and resuspended with 300 μl buffer IM-A on ice. For each fragment tested, the immobilized GST (GST-agarose) was split into a 250-μl aliquot to pre-clear the Asx in vitro translation mixture and a 50-μl aliquot for a control GST pull-down assay (30).

 Asx protein fragments were produced by in vitro translation in rabbit reticulocyte lysate (RRL; Promega). Briefly, 1 μg supercoiled pTβSTOP plasmids containing Asx DNA fragments 1.8 kb to 5 kb long (Additional file 3: Table S1) were denatured at 80 °C, chilled on ice, expressed and labeled with ^35^S using the 25-μl reaction TNT T7-coupled transcription/translation rabbit reticulocyte lysate kit (Promega). The lysates were subsequently adjusted to 5 mM Mg acetate and treated with DNase I and RNase A/RNase T1 (Fermentas) for 15 min at 25 °C, and the Roche protease inhibitor cocktail was added. The lysate was pre-cleared by mixing with 100 μl of buffer 2× PDB-P5 and 25 μl of GST-agarose for 30 min at 4 °C. To assay interactions, 65 μl of pre-cleared ^35^S-Asx-containing lysate was mixed with either immobilized 1 μg GST, GST-E(z)SET or GST-TrxSET for 2 h on a nutator at 4 °C. Lysate-bound agarose beads were washed once with 300 μl buffer 1× PDB-P5, three times with buffer WB—0.6 M NaCl, pH 7.9, and once with buffer WB—0.1 M NaCl, pH 7.9. The protein-bound agarose pellet was mixed with 15 μl 2× SDS sample buffer, resolved by SDS–PAGE and analyzed by autoradiography.

### GST pull-down western assays of embryo nuclear extract

Asx fragments indentified as interaction sites for SET domains of either Trx (TSI), E(z) (ESI) or both (ETSI) were subcloned into pGEX-6P-1, expressed and purified from Rosetta 2(DE3) cells as described above for GST-AsxETSI-2. Detailed methods are described in Additional file 5: Text S3.

### Co-immunoprecipitation western assays of embryo nuclear extract

For co-immunoprecipitation experiments, 800 micrograms of nuclear extract Bio-Rex 70 fraction was diluted into 300 μl of 1× IP buffer containing 5% polyethylene glycol 10,000 in place of Ficoll and mixed with 1:100 dilution of purified sheep anti-Asx IgG at 4 °C for 3 h [25]. Immune complexes were precipitated with protein G-Sepharose at 25 °C for 15 min and washed six times with buffer TELG containing 0.22 and 0.26 M KCl and two times with buffer TELG containing 0.05 M KCl. The samples were subsequently resolved on SDS–polyacrylamide gels and transferred onto nitrocellulose membranes for western analysis as described above. Blots were probed with 1:3000 dilution of purified rabbit anti-Trx IgG or 1:200 dilution of rabbit anti-E(z) IgG (Santa Cruz Biotech, cat# sc-98265).

### Demonstrating protein–protein interaction in situ by PLA

*Drosophila* S2 cells were cultured at room temperature in chamber slides, heat shocked at 37 °C for 15 min and allowed to recover 60 or 180 min at room temperature. Cells were fixed with 2% formaldehyde in culture medium for 20 min, washed with PBS, blocked and incubated overnight at 4 °C with either sheep anti-Asx and rat anti-Trx or sheep anti-Asx and rabbit anti-E(z). Proximity ligation assays (PLAs) were performed as described [27, 28] with several modifications. Secondary antibodies (Jackson Immuno Research) were conjugated to 5′-amino-modified MTPX oligonucleotides in Additional file 6: Table S2 using the Thunder-Link oligo conjugation systems (Innova Biosciences 420-0300) and stored at 4 °C. Circularization PLA 5′-phosphorylated oligonucleotides as well as detector PLA oligonucleotides were synthesized (Additional file 6: Table S2). For each reaction, 40 μl secondary antibodies with conjugated oligonucleotides were incubated on a shaker for 1 h in a humidity chamber at 37 °C. Three circularization PLA oligos were annealed to two corresponding PLA probes (Additional file 6: Table S2) and ligated with T4 DNA ligase (Thermo) for 30 min at 37 °C. A closed circle forms if proteins are in close proximity [29]. Rolling-circle amplification by phi28 polymerase (Thermo) was carried out in the presence of fluorescent-labeled detector oligonucleotides (Additional file 6: Table S2) for 100 min in the dark at 37 °C.

### Immunostaining of salivary gland polytene chromosomes

Preparation and immunostaining of chromosomes have been described [23]. For immunostaining, sheep anti-Asx IgG and FITC- or Texas-Red-conjugated secondary antibodies (Jackson Immunoresearch, PA) were used at dilutions of 1:100 [25]. Images of labeled chromosomes were acquired with a Zeiss microscope equipped with a digital camera, and processed using Adobe Photoshop.

### Embryo collection

Flies were acclimated to the laying chamber for 2 days before 10–14-h AEL embryos from about 300 flies were collected at 22 °C on 2% agar (supplemented with 1% sucrose/3.5% ethanol/1.5% apple cider vinegar). Embryos on laying plates were immediately washed onto a nylon sieve to remove excess yeast, dechorionated with 50% bleach and washed twice with 120 mM NaCl; 0.02% Triton X-100, followed by two washes in 1x PBS; 0.05% Triton X-100. *Asx* ^*3*^ homozygous mutant embryos were identified by the absence of *GFP* expression under a wide-field GFP fluorescence microscope. The wild-type strain Oregon R was used as a control.

### Heat-shock induction and recovery in embryos

Wild-type or homozygous *Asx* ^*3*^ mutant embryos were collected in 200 μl of 1× PBT into plastic microcentrifuge tubes that were incubated in a 37 °C water bath for 5, 10 or 15 min. To study recovery, embryos heat shocked for 15 min were transferred onto a small piece of moist filter paper placed in a moist chamber and incubated for up to 180 min at room temperature, and transferred into a new tube with 200 μl of 1× PBT for further analysis.

### Determining *hsp70* mRNA level in embryos

RNA preparation and first-strand cDNA synthesis were done as previously described [30]. A dilution series of *Drosophila* genomic DNA was used to generate standard curves for *hsp70* and *Ahcy89E*. The relative mRNA levels of genes were measured with comparison to the standard curve. All quantitative PCRs (qPCRs) were performed on the Step-One Plus Real-Time PCR system (ABI). The *hsp70* expression level in different samples was normalized to the expression of *Ahcy89E*, whose expression does not change during heat shock.

### Chromatin immunoprecipitation (ChIP)

ChIP was carried out essentially as described [31] except as follows. Exactly 200 embryos (in 200 μl buffer) were sonicated for five pulses of 10 s at 30% power at room temperature with an ultrasonic processor (CPX 130 PB, Cole Parmer), followed by 50 s on ice to yield 500-bp fragments. Samples were mixed with 200 μl of 6 M urea and incubated for 10 min on ice, and insoluble material was removed by centrifugation at 12,000×*g* for 10 min at 4 °C. The supernatant was divided into 4 × 100 μl aliquots, and ChIP experiments were performed with H3K4, H3K27 and IgG antibodies. After the final washing step, 100 μl of 10% Chelex 100 resin (BioRad) was added to protein G beads with vortexing, and samples were incubated at 95 °C for 10 min. Samples were deproteinized with proteinase K (Sigma) at 55 °C, incubated for a further 10 min at 95 °C and centrifuged to recover the beads [32]. Approximately 3% of the immunoprecipitated material was assayed by qPCR using primers specific to sequences at the *hsp70* promoter downstream, *bxd* PRE and *Ubx* promoter (Additional file 7: Text S4).

## Results

### Asx contains a bipartite site for interaction with SET domains of both E(z) and Trx

**Fig. 1:**
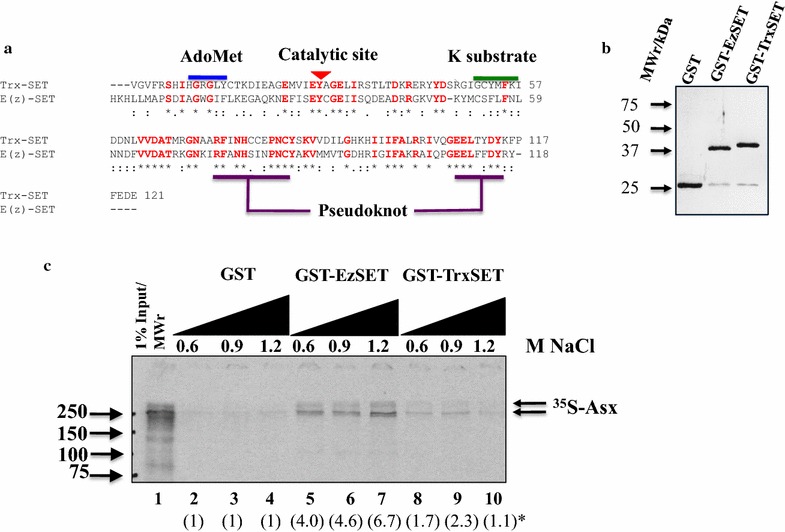
Full-length Asx interacts directly with SET domains of trxG activator, Trx and PcG repressor, E(z) in a GST pull-down assay. **a** Clustal Omega sequence alignment of SET domains of Drosophila Trx and E(z), showing 35% sequence identity. **b** SDS–PAGE (13%) analysis of affinity purified E. coli-expressed GST fusions of SET domains of E(z) and Trx. **c** GST pull-down analysis of ^35^S-methionine-labeled rabbit reticulocyte lysate-in vitro translated (RRL-IVT) Asx with GST fusions of SET domains of E(z) and Trx. The stringency of the NaCl washes indicates stronger interaction between Asx and GST-E(z)SET than GST-TrxSET. Proteins were analyzed on 13% SDS–polyacrylamide reducing gels. ()* Estimated fold binding of Asx with GST fusions relative to GST (determined by densitometry) is shown beneath each lane

Genetic analysis suggests that Asx is required for both trxG and PcG function. However, no genetic experiment can show that Asx has a direct effect on the histone methyltransferases (HMT) responsible for trimethylation of H3K4 and H3K27. Therefore, we looked for evidence of direct association of Asx with Trx, a key HMT for H3K4, and E(z), the HMT for H3K27. Alignment of the protein sequences of Trx and E(z) revealed a SET domain catalytic site [33] with a 35% amino acid identity between both proteins (Fig. 1a). To determine whether association between Asx and both HMTs can occur through the SET domains of Trx and E(z), we developed a GST pull-down autoradiography (GST pull-down) assay [34]. The SET domains of Trx and E(z) were fused to GST, expressed in *E. coli* Rosetta 2 (DE3) cells, purified by GSH-agarose affinity chromatography and immobilized on GSH-agarose (Fig. 1b). A preparation of ^35^S-Met-labeled Asx produced using coupled in vitro transcription/translation in rabbit reticulocyte lysate (IVT–RRL) (Promega) was mixed with immobilized GST-TrxSET, GST-E(z)SET and GST in control experiments. Full-length ^35^S-Asx interacted specifically with both GST-E(z)SET and GST-TrxSET (Fig. 1c) and exhibited a difference in the salt sensitivity of association with GST-TrxSET relative to GST-E(z). Higher salt concentrations increased the association of Asx with GST-E(z)SET but decreased the association with GST-TrxSET, which may reflect ionic or hydrophobic effects on interaction [35].

**Fig. 2:**
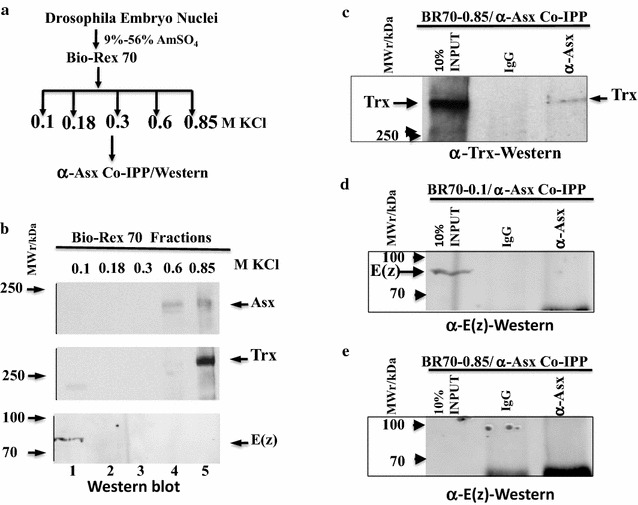
Asx co-fractionates and co-immunoprecipitates with Trx in embryo nuclear extracts. **a** Schematic of Bio-Rex-70 fractionation of embryo nuclear extracts and coupled co-immunoprecipitation–western blot analysis. **b** Western blot analysis of Bio-Rex 70 fractions with anti-Asx (upper panel), anti-Trx (middle panel) and anti-E(z) (lower panel). **c** Coupled anti-Asx immunoprecipitation and anti-Trx western blot analysis of the BR70-0.85 fraction, showing Asx association with Trx. **d** Coupled anti-Asx immunoprecipitation and anti-E(z) western blot analysis of the BR70-0.1 fraction, showing lack of Asx association with E(z) in the enriched E(z) fraction. **e** Coupled anti-Asx immunoprecipitation and anti-E(z) western blot analysis of the BR70-0.85 fraction, showing lack of Asx association with E(z) in the enriched Trx fraction

To determine the strength of association of Asx with E(z) or Trx in nuclear extracts, given that in vitro, the Asx-Trx(SET) interaction is about 5× weaker than the Asx-E(z)(SET) interaction, the Bio-Rex 70 fractions were further analyzed by anti-Asx co-immunoprecipitation coupled with α-Trx or α-E(z) western blotting (Fig. 2). E(z) and Trx were resolved into two distinct salt fractions of 0.1 and 0.85 M, respectively, by Bio-Rex 70 (Fig. 2b). Asx was co-eluted with Trx in the BR70-0.85 fraction and weakly co-immunoprecipitated at 0.26 M NaCl (Fig. 2c). By contrast, there was no detectable co-elution nor co-immunoprecipitation of Asx with E(z) in either the BR70-0.1 or BR70-0.85 fractions (Fig. 2d, e). This suggests that in nuclear extracts, the interaction between Asx and E(z) is weakly ionic or transient, which may be readily disrupted on the Bio-Rex 70 resin by a salt gradient. Alternatively, the amount of Asx in association with E(z) is below the limits of detection of immunoprecipitation. A third untested explanation is that interactions between Asx and Trx complexes are more stable than interactions of the individual proteins, and vice versa for Asx and E(z).

**Fig. 3:**
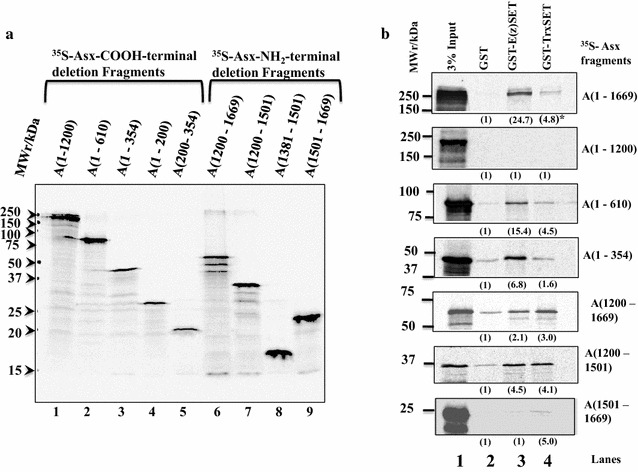
Asx contains two shared interaction sites for GST-TrxSET and GST-E(z)SET. **a** SDS–PAGE analysis of expression of ^35^S-labeled RRL-IVT deletion fragments of Asx (3% of the input for GST pull-down assay in Fig. 2b) and exposed for an hour at −70 °C (lanes 1–9). **b** Identification of two shared interaction sites for GST-E(z)SET and GST-TrxSET (1–354 and 1200–1500) and one unique, weak site for GST-TrxSET (1501–1669) on Asx. GST pull-down analysis of the Asx fragments in A) with GST-E(z)SET (lane 3) and GST-TrxSET (lane 4) compared to GST controls (lane 1). ()* Estimated fold binding of Asx with GST fusions relative to GST (determined by densitometry) is shown beneath each lane. Unbound fragments, as shown for A(1–1200), are listed in Fig. 3b

**Fig. 4:**
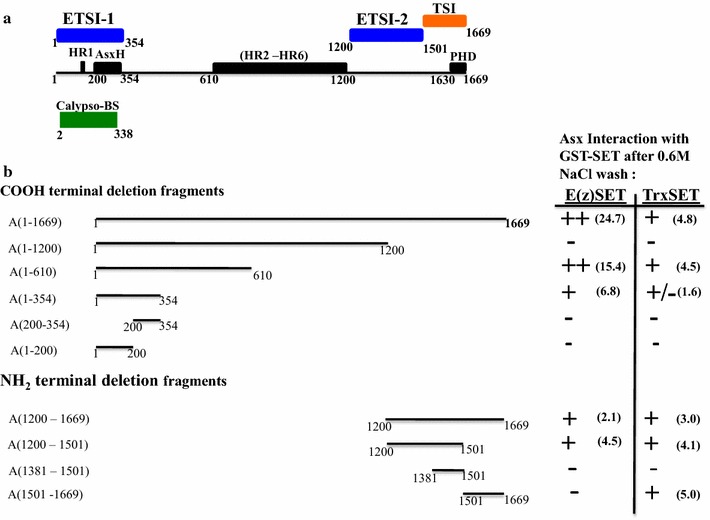
Map of Asx summarizing interaction sites of E(z) and Trx SET domains indentified using a GST pull-down assay. **a** Linear map of Asx showing a bipartite recognition site for both E(z)SET and TrxSET domains. The NH2 terminal recognition site (residues 1–354) termed E(z)-Trx SET domain interaction site 1 (ETSI-1) and the COOH terminal site (residues 1200–1501) termed ETSI-2 are marked above in blue. The single weak recognition site for TrxSET domain (residues 1501–1669), termed TSI, is marked in orange. The ETSI-1 site overlaps both the conserved AsxH domain (residues 200–354) and the Calypso binding site (marked below in green). **b** Summary of the GST pull-down analysis of the interaction between ^35^S-labeled Asx deletion fragments with GST fusion proteins of E(z) SET domain or Trx SET domain after a 0.6 M NaCl wash. Non-binding Asx fragments A(1–1200), A(1–200), A(200–354) and A(1381–1501) are indicated by (−). Scouring of the interactions of the Asx deletion fragments with GST-E(z) SET or GST-Trx SET is based on the densitometry of band intensity relative to GST [(++; > tenfold),; +; (twofold–tenfold)]

The interaction sites of GST-E(z)SET and GST-TrxSET on full-length Asx were mapped by determining the association of ^35^S-Asx COOH terminal or NH_2_ terminal deletion fragments (Fig. 3a) with GST-E(z)SET and GST-TrxSET using the GST pull-down assay (Figs. 3b, 4). Two interaction sites were identified on Asx for both GST-E(z)SET and GST-TrxSET (Fig. 3b): (1) at NH_2_ terminal residues 1–354 termed E(z)/Trx SET domain interaction site 1 (AsxETSI-1) and (2) at COOH terminal residues 1200–1501 termed (AsxETSI-2) (Fig. 4). In addition, a weak but specific COOH interaction site at terminal residues 1501–1669 was mapped for Trx (Fig. 3b), termed Trx SET domain interaction site (AsxTSI) (Fig. 4). These results suggest that Asx contains a bipartite E(z)/Trx SET domain interaction (ETSI-1 and ETSI-2) site that has low sequence identity (14.97%) and is separated by 846 amino acids (Additional file 8: Fig. S2). It is interesting that the Asx NH_2_ terminal residues 1–1200 which contain ETSI-1 did not show any significant binding with GST-E(z)SET and GST-TrxSET. In together with other pull-down results, it is possible that the Asx 610–1200 fragment which contains HR2–HR6 domain plays a negative role on ETSI-1 and GST-E(z)SET/GST-TrxSET interaction.

**Fig. 5:**
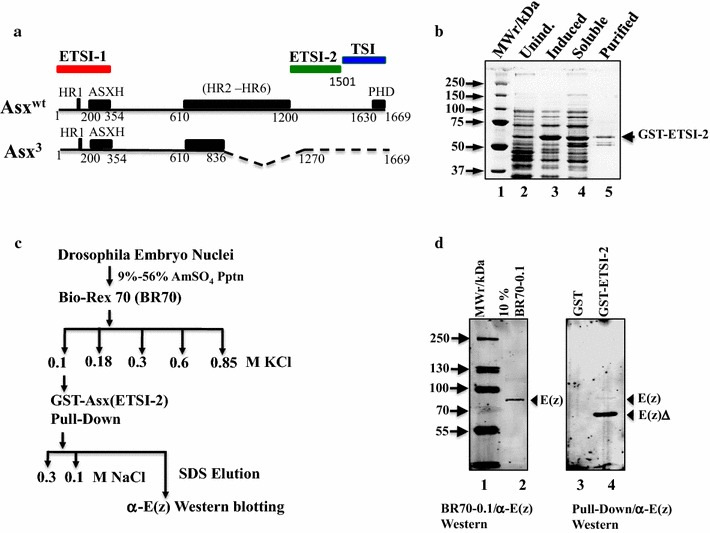
GST-fused AsxETSI-2 interacts with E(z) in embryo nuclear extracts. **a** Alignment of the amino acid sequence of wt and Asx^3^ mutant indicates deletion of ETSI-2 and TSI sites in the COOH terminal region, which may disturb the levels of H3K4me3 and H3K27me3. **b** SDS–PAGE (13%) analysis of purification of *E*. *coli*-expressed GST fusions of ETSI-2 domains. **c** The interaction of ETSI-2 with endogenous E(z) was assayed using extracts prepared from 6- to 18-h-old embryo nuclei. Fractionation of extracts on a Bio-Rex70 column by a step gradient from 0.1 to 0.85 M NaCl elutes E(z) at 0.1 M NaCl fraction as opposed to Trx which elutes at 0.85 M NaCl. **d** GST-fused ETSI-2 was mixed with the 0.1 M NaCl-containing E(z) fraction in a pull-down assay, using GST as a negative control. The pull-down products were analyzed by 7.5% SDS–PAGE and probed by anti-E(z) antibody in a western blot assay. Lanes (1) pre-marked molecular weight markers (BioRad); (2) 10% input of the pull-down fraction; (3) GST/BR70-0.1 fraction pull down; (4) GST-ETSI-2/BR70-0.1 fraction pull down. The lower band in lane 4 is likely a breakdown product of E(z)

Alignment of the amino acid sequences of *Asx* ^*3*^ mutants and wild type [25] indicated a deletion of the COOH terminal ETSI-2 and TSI in *Asx* ^*3*^ mutants (Fig. 5a). To test for interaction between E(z) and AsxETSI-2, we developed a GST pull-down western assay using purified GST-ETSI-2 (Fig. 5b) and chromatography fractionated embryo nuclear extracts as a source of E(z) (Fig. 5c). Similar experiments were not performed with AsxETSI-1 because of the instability of purified GST-ETSI-1. The E(z)-enriched BR70-0.1 M chromatography fraction (Fig. 5c) was mixed with immobilized GST-ETSI-2 and resolved by SDS–PAGE coupled with western blotting using anti-E(z) antibody. Two bands were specifically detected on the blot corresponding to E(z) and a breakdown fragment (Fig. 5d) consistent with the interaction of GST-E(z) with AsxETSI-2. It is possible that E(z) was fragmented due to the sub-optimized pull-down condition. These results indicate that Asx ETSI-2 (which is deleted in *Asx* ^*3*^) can associate with E(z) in vivo.

### Asx associates with E(z) and Trx after induction and recovery from heat shock in vivo

**Fig. 6:**
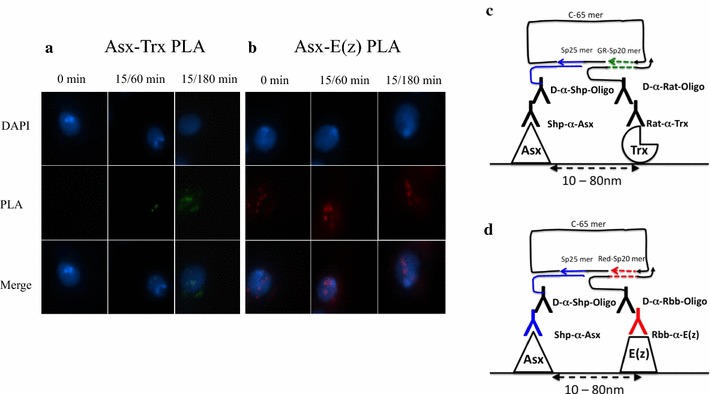
Asx interacts with E(z) and Trx in *Drosophila* S2 cells in vivo. **a** Proximity ligation assay (PLA) between Asx and Trx with *Drosophila* S2 cells before and after heat-shock induction. Nuclei were labeled with DAPI in blue and PLA signals in green for Asx-Trx and in red for Asx-E(z). **b** PLA between Asx and E(z) with S2 cells before and after heat-shock induction. **c**, **d** Schematic diagram of PLA reaction. Oligonucleotides and antibodies used in PLA are listed in “Methods” section. A closed circle between connector and splints forms if proteins are in close proximity. Rolling-circle amplification (RCA) was carried out in the presence of fluorescent-labeled oligonucleotides. Signals would be detected only when proteins were in distance between 10 and 80 nm

To further investigate whether Asx weakly or transiently associates with Trx and E(z) in vivo, we performed proximity ligation assay (PLA) in situ following heat shock on *Drosophila* S2 cells. This assay allows very sensitive (compared to immunoprecipitation) detection of protein–protein interactions either in solution or in situ at single-cell resolution [36]. Association between Asx with Trx was only observed after heat-shock induction, and the level of association becomes significant after 60–180 min of recovery period (Fig. 6a). The association between Asx with E(z) was observed before and after heat-shock induction (Fig. 6b). These results suggest Asx associates with E(z) and Trx in vivo during heat-shock recovery. Taken together, these three protein assays indicate that Asx associates both in vitro and in vivo with E(z) and Trx.

### Asx binds to *hsp70* promoter downstream region upon heat-shock induction and is required for *hsp70* repression during induction and recovery

**Fig. 7:**
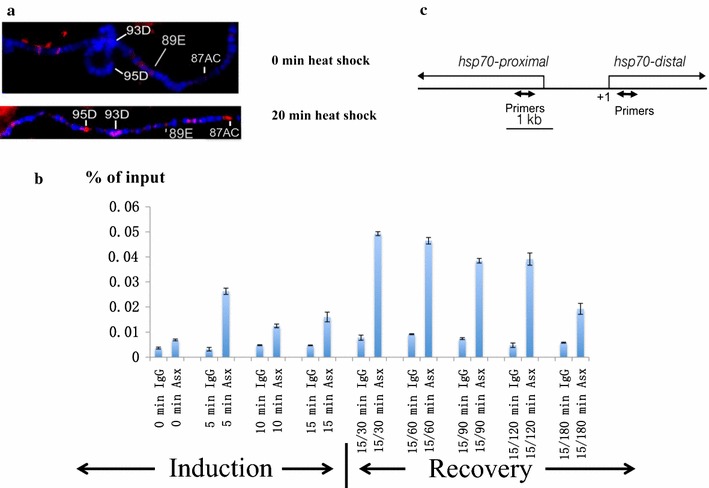
Asx binds to the *hsp70* promoter downstream upon heat-shock induction. **a** Asx antibody staining of the central part of wild-type 3R polytene chromosomes before and after 20-min heat shock at 37 °C. The major heat-shock loci at 87AC, 93D and 95D are labeled. After 20-min heat shock, Asx is recruited to major heat-shock loci indicated. The bithorax complex located at 89E serves as a control for constitutive binding of Asx. **b** Chromatin immunoprecipitation with anti-Asx and IgG control antibodies from wild-type embryos during heat-shock induction at 37 °C and recovery. The DNA from ChIP samples at *hsp70* promoter downstream region was analyzed by qPCR and is shown along the *y*-axis, and times of heat shock or recovery are shown in the *x*-axis. Duration of heat shock/recovery is denoted as 15/30, 15/60, 15/90, 15/120 and 15/180 min. The signals are represented as mean ± SEM with *N* = 3. **c** Primer map showing the location of primers 218- and 392-bp downstream of *hsp70* transcription start site used in ChIP experiments

The foregoing results suggest a mechanism whereby Asx modulates the ratio of H3K4 and H3K27 trimethylation by associating with the antagonistic HMTs during heat shock and recovery. If so, then mutations in *Asx* should affect levels of trimethylation at histone H3K4 and H3K27 during transcription. To determine whether Asx was recruited to heat-shock loci on chromatin, polytene chromosomes were prepared from salivary glands subjected to 20 min of heat shock at 37 °C, and stained with antibodies to Asx. These were compared to preparations from glands that were not heat shocked. Asx was recruited to *hsp70* region (at 87AC) and other heat-shock loci following 20 min of heat shock (Fig. 7a). In contrast, in control polytene chromosomes that were not heat shocked, Asx was recruited to region 89E (Fig. 7a) which includes *Ultrabithorax* (*Ubx*) thus serving as a positive control for the polytene staining [25].

Pol II recruitment and initiation of *hsp70* transcription to generate the paused *hsp70* promoter occur early in embryogenesis (2–4 h after egg deposition) when maternally deposited Asx protein or *Asx* mRNA is still present. To allow enough time for maternal levels of *Asx* mRNA or protein to drop, and thus allow us to detect the embryonic effect of *Asx* mutations, heat shock was induced in 14–16-h embryos. Using this protocol, we are unable to assay the role of *Asx* in establishment of transcriptional initiation and promoter clearance of *hsp70* in early embryos. Our studies in later embryos do not attempt to distinguish the role of *Asx* in elongation between roles at the promoter in re-initiation and promoter clearance, although we biased the results toward elongation by selection of a primer downstream of the promoter (+218 to +392 bp) previously identified as a site of recruitment for Trx upon heat shock [23] for the ChIP experiments below.

The *hsp70* gene is induced in all cells of the embryo in response to thermal stress, allowing us to use whole embryos for this study [37]. To determine whether Asx binds downstream of the *hsp70* promoter downstream region, we performed ChIP and quantitative PCR (qPCR) using the anti-Asx antibody (Fig. 7b, c). In control embryos that were not subject to thermal stress (0 min of heat shock), Asx did not significantly bind at the *hsp70* promoter downstream region. After 15 min of heat shock, the level of Asx binding was threefold higher than with no heat shock. At 30-min recovery after 15-min heat shock, Asx binding to the *hsp70* gene increased sevenfold relative to no heat shock. The level of Asx binding at *hsp70* gradually decreased from 60-min recovery to 180-min recovery (Fig. 7b). As a positive control for Asx binding in our assay conditions, we tested Asx binding to selected regions within *Ubx* promoter and *bithoraxoid* (*bxd*) Polycomb response element (PRE) in *Drosophila* embryos with the our Asx antibody in our ChIP assay [10]. The results were consistent with previous observations that Asx binds chromatin at these locations without heat-shock induction (Additional file 9: Fig. S3). These results confirm that the *hsp70* locus is a direct binding target of Asx upon heat shock, with peak binding occurring between 15-min induction/30-min recovery and 15-min induction/120-min recovery.

**Fig. 8:**
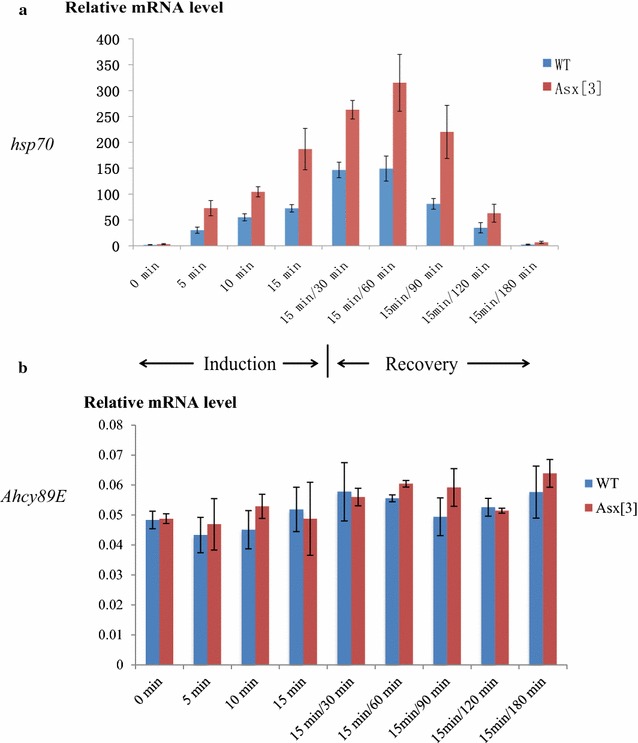
*Asx* is required for *hsp70* repression during heat-shock induction and recovery. **a** The relative mRNA levels in wild-type and *Asx* ^*3*^ homozygous null mutant embryos were measured by RT-qPCR. *Asx* null mutations are embryonic lethal at a late embryonic stage [4], so homozygous 12–15-h *Asx* ^*3*^ mutants were collected using absence of expression of the GFP-marked balancer chromosome as a criterion. The duration of heat shock/recovery is as described in Fig. 7. The *x*-axis shows the heat-shock induction times at 37 °C and heat-shock recovery times after 15 min of 37 °C heat-shock induction. The *y*-axis indicates the *hsp70* mRNA level normalized to the control gene *Ahcy89E* mRNA level. The signals are represented as mean ± SEM with *N* = 3. **b** The relative mRNA levels of the control gene *Ahcy98E* are compared between *Asx* ^*3*^ mutant and wild-type embryos throughout the heat-shock induction and recovery. The *x*-axis shows the heat-shock induction times at 37 °C, and heat-shock recovery times after 15 min of 37 °C heat-shock induction. The *y*-axis shows the relative mRNA level of the control gene *Ahcy89E* mRNA, as mean ± SEM with *N* = 3

To investigate the effect of *Asx* on transcription during induction and recovery from heat shock, we compared the steady-state *hsp70* mRNA levels during heat shock and recovery of homozygous *Asx* ^*3*^ null mutants to wild-type (Oregon R) embryos (see “Methods” section). The mRNA level difference between the homozygous *Asx* ^*3*^ mutant and wild type was at least twofold during heat-shock induction and recovery, and at 90-min recovery, the difference reached a maximum level at 2.7-fold (Fig. 8a). The steady-state *Ahcy89E* mRNA levels during heat shock and recovery were stable and the mRNA level was comparable between *Asx* ^*3*^ mutant and wild-type embryos, indicating that *Ahcy89E* is suitable for normalizing the *hsp70* mRNA levels (Fig. 8b). Together, these data show that *Asx* represses the *hsp70* locus during heat-shock induction and recovery. After 180-min recovery, the *hsp70* mRNA level in both wild type and homozygous *Asx* ^*3*^ mutant decreased to the level before heat-shock induction, showing that *Asx* is not required for terminating transcription after heat-shock induction. In addition, peak transcription observed between 15-min induction/30-min recovery and 15-min induction/90-min recovery correlates with Asx binding within this period (Figs. 7b, 8a).

### *Asx* transiently reduces H3K4 trimethylation at *hsp70* during heat-shock induction

**Fig. 9:**
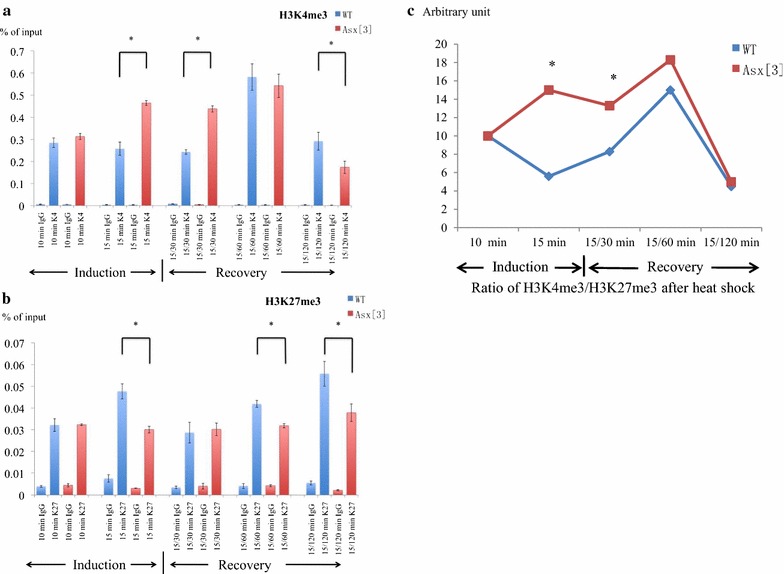
*Asx* regulates H3K4me3 and H3K27me3 levels at *hsp70*. Both panels show ChIP-qPCR analysis comparing control rabbit IgG to trimethylated histones in wild-type (blue) and *Asx* ^*3*^ (red) embryos at different times of heat-shock induction and recovery as indicated in the *x*-axis. The *y*-axis indicates recovery after ChIP as a percentage of input DNA. The notation for the duration of heat shock/recovery is described in Fig. 7. All data are represented as mean ± SEM with N = 3. (*) *P* < 0.05. The standard deviation (SD) of each data point is presented in Additional file 12: Table S3 and Additional file 13: Fig. S6. **a** ChIP-qPCR analysis with trimethylated histone H3K4 antibody. During late heat-shock induction and the first 30 min of recovery, H3K4me3 levels are higher in *Asx* mutants than wild type. In late recovery, H3K4me3 levels are lower in mutants than wild type. **b** ChIP-qPCR analysis with trimethylated histone H3K27 antibody. Levels of H3K27me3 are essentially constant in *Asx* mutants and are significantly lower than wild type at the end of induction and the recovery phases. **c** Ratio of histone H3K4me3 to H3K27me3 during heat shock and recovery, expressed in arbitrary units, and derived from the data in **a**, **b**

*Asx* represses *hsp70* during heat shock, so in *Asx* ^*3*^ mutant embryos, we expected to observe increased levels of H3K4me3 downstream of the *hsp70* promoter after heat shock compared to wild type. As shown in Fig. 9a, in ChIP-qPCR experiments, the level of H3K4 trimethylation in wild type and *Asx* ^*3*^ mutant did not differ in the first 10 min of heat-shock induction. However, at 15-min induction and 15-min/30-min recovery (Fig. 9a), the level of H3K4 trimethylation in *Asx* ^*3*^ mutants was 1.8-fold higher than in wild-type (significant at *P* < 0.05), suggesting that in wild-type embryos, *Asx* reduces the level of histone H3K4me3 at these time points. Interestingly, in the later recovery phase, *Asx* ^*3*^ mutants showed less H3K4me3 than wild-type embryos after 120 min of recovery from 15-min heat-shock induction (significant at *P* < 0.05), consistent with the rapid drop of *hsp70* mRNA level during 120-min recovery after heat-shock induction. At this time, Asx could transiently modulate the rate of transcription by governing the rate of transcript decay. We suggest that Asx directly or indirectly regulates H3K4me3 to an appropriate level during heat shock and recovery.

### *Asx* regulates the ratio of histone H3K4 to H3K27 trimethylation at *hsp70* during heat-shock induction and recovery

If Asx represses *hsp70* by acting as a PcG protein, then we expect to observe reduced H3K27me3 levels in *Asx* ^*3*^ mutants compared to wild type during heat-shock induction and recovery. We performed ChIP-qPCR experiments with anti-H3K27me3 antibody on wild-type and *Asx* ^*3*^ mutant embryos during heat shock and recovery. The level of H3K27me3 in wild type and *Asx* ^*3*^ mutant did not differ markedly at 10 min of heat-shock induction. At subsequent time points from 15-min heat-shock induction to 120-min recovery after heat shock except the 30-min recovery, the level of H3K27me3 in *Asx* ^*3*^ mutants was 1.5-fold lower than in wild type (Fig. 9b).

Thus, the data in Fig. 9a, b show that mutation of *Asx* alters the levels of H3K4me3 and H3K27me3 in the expected way at one target gene during the same regulatory event. Interestingly, the significant increase in the ratio of H3K4me3/H3K27me3 from 15-min induction to 15-min induction/30-min recovery from heat shock in *Asx* ^*3*^ compared to wild type (Fig. 9c) occurs close to the peak of transcription (Fig. 8a) that coincides with the transition from promoter clearance to elongation. As positive and negative controls for the levels of H3K4me3 and H3K27me3 in our assay conditions, we compared the highest and lowest levels of recovery of H3K4me3 and H3K27me3 observed at *hsp70* in ChIP experiments to levels obtained with a fragment from within the *bxd* PRE as a positive control, and a region upstream of the *DRP12* gene as a negative control, in *Drosophila* embryos (Additional file 10: Fig. S4). The results show that significantly more H3K4me3 is recovered at the *hsp70* locus compared to *bxd*, consistent with *hsp70* being actively transcribed in all cells, whereas *bxd* is expressed only in a subset of cells. During the recovery phase from heat shock, when H3K27me3 levels are highest, they are equivalent to those observed at *bxd*. Even at the lowest levels of H3K27me3 observed at *hsp70* (15 min of heat shock, 120 min of recovery), the levels observed are significantly higher than the negative or IgG controls. Thus, we are confident that the changes we observe in H3K4me3 and K3K27me3 we observe at *hsp70* are biologically important.

## Discussion

Our experiments on the role of *Asx* in regulation of the relative levels of H3K4me3 to H3K27me3 suggest that Asx recruits Trx and E(z) at different temporal stages via interaction with their SET domains (Figs. 9, 6). The mapping data shown in Figs. 1 and 2 confirm the hypothesis that Asx interacts directly with E(z) and Trx in vitro. The mapping data also show that there are two non-overlapping Asx domains that interact with E(z) and Trx SET domains, termed ETSI-1 and ETSI-2 (Fig. 4). Sequence comparison of ETSI-1 and 2 reveals 15% sequence identity (Additional file 8: Fig. S2), suggesting that either there is conservation of 2D structure with clustered amino acid sequence conservation, or that ETSI-1 and 2 domains have a unique interaction mechanism. The third Asx interaction site, TSI (Figs. 3, 4), is a distinct Trx SET domain interaction site, which overlaps the conserved C-terminal atypical PHD motif, consistent with the suggestion that the conserved atypical PHD motif lacks structural features which restricts its binding to the N-terminal tail of H3 [38].

The identification of Asx interaction sites for the SET domains of E(z) and Trx (ETSI-1/2 and TSI) (Fig. 4) is consistent with Asx association with equivalent levels of E(z) and Trx in vivo at 60 to 180 min of recovery (Fig. 6). These findings support the hypothesis that *Asx* directly regulates H3K4me3 and H3K27me3 levels downstream of the *hsp70* promoter (Fig. 9). However, the mapping of 2 SET domain interaction sites suggests that it is unlikely that Trx and E(z) compete to bind Asx. This conclusion is supported by the data in Fig. 2 showing that E(z) and Trx do not co-fractionate after chromatography of nuclear extracts on Bio-Rex 70. These results suggest that Asx association with Trx or E(z) in vivo may be influenced by the different temporal stages of heat shock and recovery. These experiments do not rule out interactions of Asx with domains outside the SET domains of Trx and E(z), but this possibility was not tested.

Comparison of existing structure function analysis of Asx homologs in *Drosophila* and mammals with our new data allows us to speculate that Asx acts as an ETP by interacting with multiple nucleosome-modifying enzymes to modulate histone modifications. Asx and its mammalian homolog ASXL1 interact with Calypso/BAP1 to form the PR-DUB complex, which deubiquitinates histone H2AK118Ub/H2AK119Ub at the *Ubx* PRE and promoter [10]. The ASXH domain is conserved between all mammalian ASXL and *Drosophila* Asx and directly binds the deubiquitinase BAP1 [10, 39]. Interestingly, in mammals the ASXL1 DEUBAD domain (amino acid 238–290) located within the ASXH (amino acid 249–368) domain is required to activate the BAP1 enzymatic function on deubiquinating H2AK119Ub [40, 41]. Unlike *Asx*, mutation of *calypso* did not have a significant effect on the relative *hsp70* mRNA levels in *calypso* ^*2*^ mutant and wild-type embryos at selected time points of heat shock or recovery (Additional file 11: Fig. S5). Thus, *calypso* is not required for the activity of Asx at *hsp70* during heat-shock induction and recovery.

Both our results on Asx and recent studies on ASXL1 suggest that ETSI-1 site alone is insufficient to maintain the normal H3K27me3 level at target promoters [42]. We therefore suggest that both the ETSI-1 and ETSI-2 regions are required to recruit and/or retain the function of E(z)/EZH2 at target promoters. Consistent with this view, ASXL1 co-immunoprecipitates with the PRC2 components EZH2, SUZ12 and EED [43]. The C-terminal region of ASXL1 aligns to the Asx ETSI-2 with 15% sequence identity (Additional file 8: Fig. S2B), suggesting conservation of function. It is possible that ETSI-1 and ETSI-2 interact independently or cooperatively. In the future, it will be interesting to determine whether each of these domains simultaneously recruits Trx and E(z), whether binding of Trx or E(z) occurs preferentially to ETSI-1 or 2 and whether histone demethylases or histone acetyl transferases also associate with these domains.

One major finding in this study is that *Asx* is required to repress the transcription of *hsp70* during heat stress and recovery. In the first 10 min of heat stress, when transcriptional elongation should predominate over re-initiation, *Asx* mutants exhibit higher levels of *hsp70* transcription compared to controls (Fig. 8), so *Asx* acts as a governor to prevent rapid increase in the rate of *hsp70* transcription. Interestingly, we do not detect any significant change in H3K4me3 levels during this time (Fig. 9), implying that the *Asx* effect in the first 10 min is Trx independent [23]. Given previous observations that Asx is not recruited to polytene chromosomes in *trx* mutants and vice versa [44] and that *trx* mutations abolish induction of heat shock [23], one would predict no heat-shock response in *Asx* mutants. As noted in “Background,” we would not detect a Trx-dependent effect in our experiments immediately after heat-shock induction because recruitment of Trx and Pol II and initiation of transcription occur many hours earlier in the presence of maternal Asx. We suggest that changes in H3K4me3 levels detected after 15 min of heat stress reflect re-initiation of *hsp70* transcription.

*Asx* may have a previously unreported role in transcriptional elongation of *hsp70* that is independent of the catalytic activity of Trx or other H3K4 HMT because *Asx* mutants have higher transcription compared to wild type in the first 10 min of heat stress that cannot be attributed to changes in levels of H3K4me3. *Asx* may regulate factors required for *hsp70* transcriptional elongation including Mediator, elongation factors (such as Spt5, Spt6 and FACT) or the H3K36 histone methyltransferase activity [18, 20, 22, 45]. Alternatively, *Asx* may participate in Pol II pausing and retention or release during the 0–10-min heat-shock phase before a Trx-dependent step [46].

From 15 min of heat-shock induction up to 60 min of recovery, *Asx* is required to govern the rate of *hsp70* transcription and changes the ratio of H3K4me3 to H3K27me3. As shown in Fig. 9c, the ratio of H3K4me3/H3K27me3 levels is consistently higher in *Asx* mutants compared to wild type during this period suggesting that the relative levels of H3K4 and H3K27 trimethylation regulate *hsp70* transcription. Alternatively, the ratio of H3K4me3/H3K27me3 may reflect consequences of other events regulated by *Asx* that lead to changes in the ratio.

From 90 to 180 min after recovery from heat shock, transcription of *hsp70* falls in both control and *Asx* ^*3*^ mutant embryos, showing that *Asx* is not required for repression of *hsp70* transcription at this phase (Fig. 8a). By contrast, the PcG gene *pho* is required for this repression because the level of *hsp70* mRNA in *pho* ^*1*^ mutant larvae was significantly higher than in wild-type embryos after 30-min induction/180-min or 300-min recovery, but no significant difference was observed after 30-min induction/60-min recovery after heat shock [24]. Therefore, the timing of Asx regulation of *hsp70* transcription does not significantly overlap with the requirement for *pho*.

*Asx* was identified first as a PcG gene [4], and subsequent experiments in *Drosophila* supported this conclusion [5, 6]. To our knowledge, our observations provide the first example where *Asx* mutations cause simultaneous changes in both H3K4me3 and H3K27me3 at the same locus at the same time. Protein nulls such as *Asx* ^*22P4*^ have no change in global H3K27 trimethylation and show very slight reduction in global H3K4 trimethylation [10]. The increased sensitivity of gene-specific analysis or particular features of *hsp70* regulation may allow us to detect clear effects of *Asx* mutations on relative levels of H3K4me3 and H3K27me3 that are not detected in bulk chromatin. Our data do not rule out alternative models could account for the regulation of the H3K4me3 to H3K27me3 levels: (1) Asx acts indirectly via CBP-mediated H3K27 acetylation to block methylation [47, 48]; (2) a role for two other H3K4me3 HMTs, SET1 and Trr, cannot be excluded since both contain SET domains with 50% amino acid sequence identity to the SET domain of Trx (NCBI protein sequences, see web refence below); (3) Asx affects demethylation of H3K27me3 by Jarid2 by recruiting E(z), and its HMT PRC2 subunits Su(z)12, Esc and Jarid2 [49].

## Conclusions

The major finding in this study is that Asx interacts directly with E(z) and Trx in vitro and in vivo during heat-shock recovery. *Asx* is required to repress transcription of the *hsp70* locus during heat stress and the first 60 min of recovery by regulating the relative H3K4 and H3K27 trimethylation levels at the promoter. These results are consistent with genetic identification of *Asx* as an ETP required for both trxG and PcG function.

## Additional files

**Additional file 1: Text S1.** Rabbit anti-Asx antibody.

**Additional file 2: Fig. S1.** Validation of Asx antibody. Western blots with *Drosophila* wild-type and *Df(2R)trix* mutant embryo extract showing the rabbit anti-Asx antibody (aa. 200–356) generated for this study binds specifically to Asx. *Df(2R)trix* mutant contains deletion of entire *Asx*. The binding level was significantly reduced in *Df(2R)trix* mutant embryo extract compared to wild-type embryo extract.

**Additional file 3: Table S1.** PCR primer pairs for construction of E(z) SET and Asx expression vectors.

**Additional file 4: Text S2.** Buffers.

**Additional file 5: Text S3.** GST pull-down western assays of embryo nuclear extract.

**Additional file 6: Table S2.** Oligonucleotides for *in situ* PLA in *Drosophila* S2 cells.

**Additional file 7: Text S4.** Primers for *hsp70*, *Ahcy89E*, *bxd* PRE and *Ubx* promoters.

**Additional file 8: Fig. S2.** Alignment of amino acid sequences of AsxETSI. **(A**) Clustal Omega alignment of AsxETSI-1 and AsxETSI-2 showing 14.97 % sequence identity. **(B**) Clustal Omega alignment of AsxETSI-2 and ASXL1 (943–1307) showing 15.1% sequence identity.

**Additional file 9: Fig. S3.** Asx binds to *Ultrabithorax* (*Ubx*) promoter and *bithoraxoid* (*bxd*) PRE region. (**A)** Primer map showing the location of primers used in ChIP experiments. L2, L7 and L8 primers are located within the *bxd* PRE region, 12.5-kb upstream of the *Ubx* promoter. U2 and U3 primers are located downstream of the *Ubx* promoter. **(B, C)** ChIP-qPCR analysis of anti-Asx and control rabbit IgG antibodies from wild-type embryos. The DNA recovered from ChIP samples was analyzed by qPCR and is shown along the *y*-axis. The signals are represented as mean ± SEM with N = 3.

**Additional file 10: Fig. S4.** H3K4me3 and H3K27me3 levels at *bithoraxoid* (*bxd*) Polycomb response element (PRE) and DPR12 genes compared to highest and lowest levels observed at *hsp70*. ChIP-qPCR analysis of H3K4me3 and H3K27me3 and control rabbit IgG antibodies from wild-type embryos. The DNA recovered from ChIP samples was analyzed by qPCR, and percent recovery is shown along the *y*-axis. The data for *hsp70* are taken from Fig 9. The signals are represented as mean ± SEM with N = 3. The *bxd* PRE primers are located between BX-C 218839 and 218959. C1 is located at +39kb to the DPR12 gene.

**Additional file 11: Fig. S5.** *calypso* is not required for *hsp70* repression during heat-shock induction and recovery. The relative mRNA levels in wild-type and *calpyso* ^*2*^ homozygous null mutant embryos were measured by RT-qPCR. The *x*-axis shows the heat-shock induction times at 37 °C, and heat-shock recovery times after 15 min of 37 °C heat-shock induction. The *y*-axis indicates the *hsp70* mRNA level normalized to the control gene *Ahcy89E* mRNA level. The signals are represented as mean ± SEM with N = 3.

**Additional file 12: Table S3.** Standard deviation (SD) table for ChIP experiments.

**Additional file 13: Fig. S6.** Asx regulates H3K4me3 and H3K27me3 levels at *hsp70*. Both panels show ChIP-qPCR analysis comparing control rabbit IgG to trimethylated histones in wild-type (blue) and *Asx* ^*3*^ (red) embryos at different times of heat-shock induction and recovery as indicated in the x-axis. The y-axis indicates recovery after ChIP as a percentage of input DNA. The notation for the duration of heat shock/recovery is described in Fig. 7. All data are represented as mean ± SD. There was minimal difference when compared with the error bars using SEM as the error source in Fig. 9.

## Abbreviations

PcG: Polycomb group
TrxG: trithorax group
ETP: enhancer of trithorax and Polycomb
Asx: additional sex combs
HMT: histone methyl transferase
E(z): enhancer of zeste
Trx: trithorax
hsp70: 70-kDa heat-shock proteins
SET: Su(var)3–9, enhancer of zeste and trithorax
CTD: C-terminal domain
HSF: heat-shock factor
ChIP: chromatin immunoprecipitation
ETSI: E(z)/Trx SET domain interaction site
TSI: Trx SET domain interaction site
PLA: proximity ligation assay
Ahcy89E: adenosylhomocysteinase 89E
PR-DUB: Polycomb repressive deubiquitinase
