# Supplementary material for: Additional sex combs interacts with enhancer of zeste and trithorax and modulates levels of trimethylation on histone H3K4 and H3K27 during transcription of hsp70
Source: Epigenetics Chromatin. 2017 Sep 19;10:43. doi: 10.1186/s13072-017-0151-3 (PMC5605996; doi:10.1186/s13072-017-0151-3)
Supplement: Supplementary file 1 — Additional file 1: Text S1. Rabbit anti-Asx antibody. [file 13072_2017_151_MOESM1_ESM.docx]

**Text S1** Rabbit anti-Asx antibody

The *Drosophila* Asx aa 200 - 356 region was amplified by PCR with primer pairs: forward 5’-ccggttaCAT ATG GCG CAG ATT GAG C-3’ and reverse 5’-ccggtatCCC GGG TCA CTT CTG ATT CTT GCA AT C-3’ and subcloned into the *Nde*I - *Sma*I site of pGEX-6P-1 (GE Healthcare) to generate a GST-Asx fusion and expressed in *E. coli* Rosetta 2(DE3) strain (Novagen). The fusion protein was solubilised in 8 M urea/5 mM EDTA/720 mM 2-mercaptoethanol/PBS, pH 7.4, diluted to 1 M urea/5 mM EDTA/10 mM 2-mercaptoethanol/PBS, pH 7.4 and purified on GSH–Agarose (Sigma) using standard protocols. The purified protein was dialysed into 1 M urea/10 mM 2-mercaptoethanol/PBS, pH 7.4 prior to immunization. Protein concentrations were determined by the Bradford Assay (Thermo) using BSA as a protein standard. After three boosts, rabbit anti-Asx antisera was collected and stored at -80^o^C.
