# Supplementary figures and images for: Additional sex combs interacts with enhancer of zeste and trithorax and modulates levels of trimethylation on histone H3K4 and H3K27 during transcription of hsp70
Source: Epigenetics Chromatin. 2017 Sep 19;10:43. doi: 10.1186/s13072-017-0151-3 (PMC5605996; doi:10.1186/s13072-017-0151-3)

Figure S1

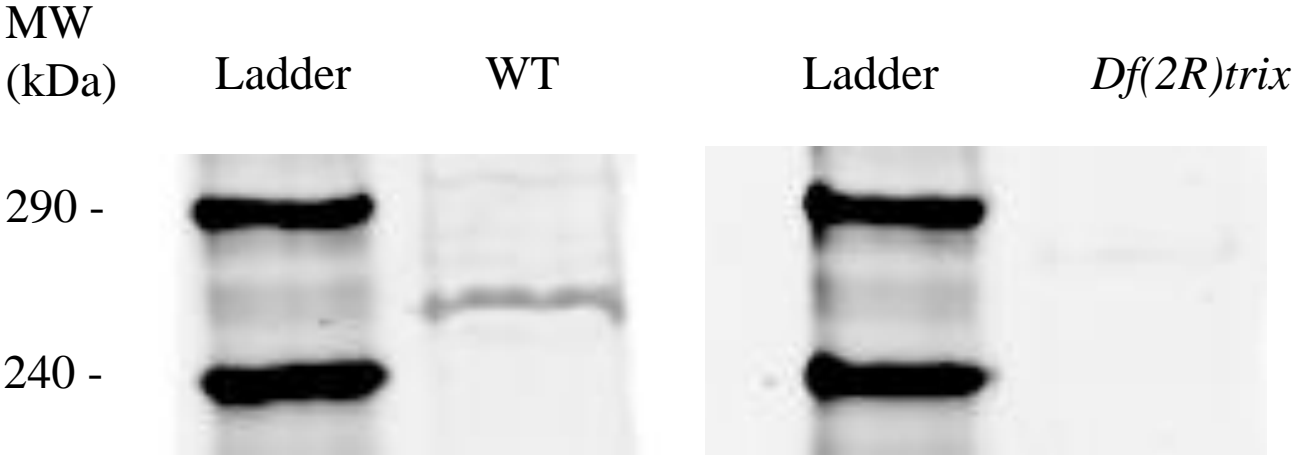

Supplement: Supplementary file 2 — Additional file 2: Fig. S1. Validation of Asx antibody. Western blots with Drosophila wild-type and Df(2R)trix mutant embryo extract showing the rabbit anti-Asx antibody (aa. 200–356) generated for this study binds specifically to Asx. Df(2R)trix mutant contains deletion of entire Asx. The binding level was significantly reduced in Df(2R)trix mutant embryo extract compared to wild-type embryo extract. [file 13072_2017_151_MOESM2_ESM.pdf]

Figure S3

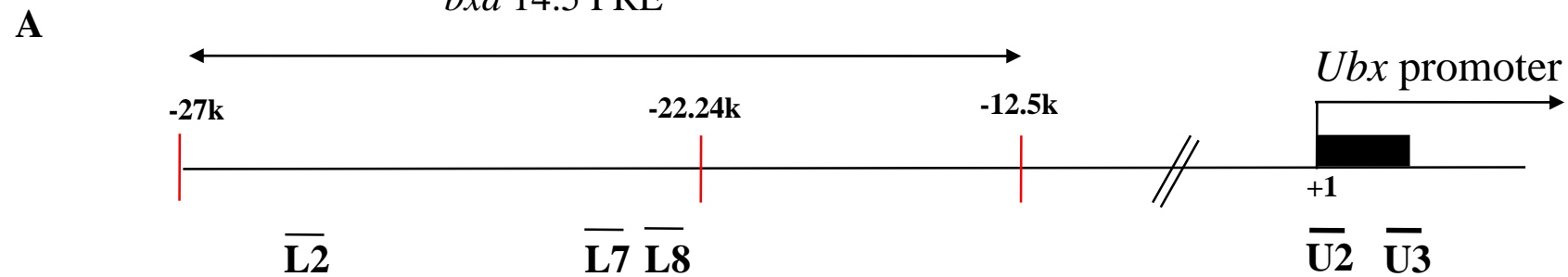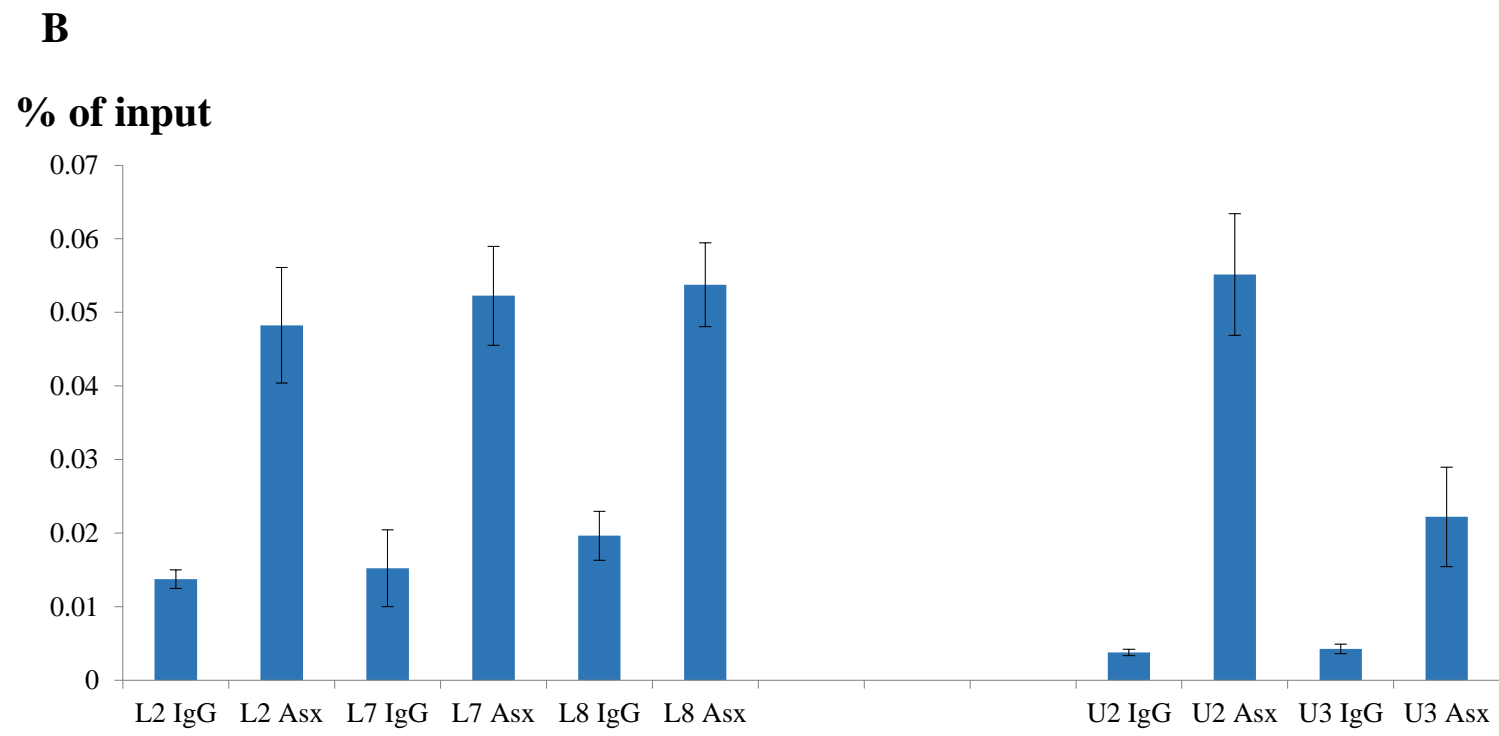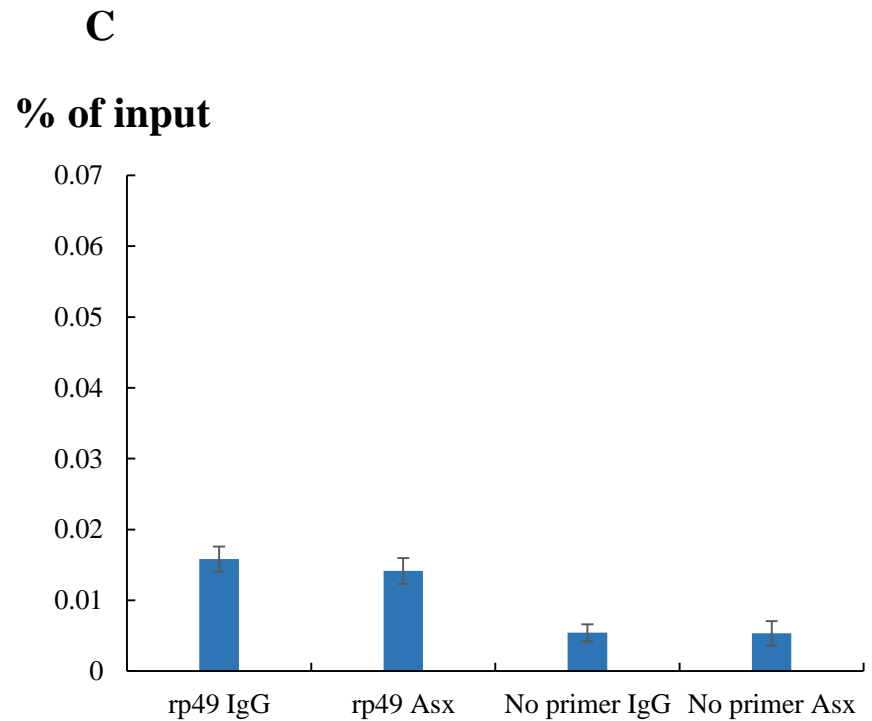

Supplement: Supplementary file 9 — Additional file 9: Fig. S3. Asx binds to Ultrabithorax (Ubx) promoter and bithoraxoid (bxd) PRE region. (A) Primer map showing the location of primers used in ChIP experiments. L2, L7 and L8 primers are located within the bxd PRE region, 12.5-kb upstream of the Ubx promoter. U2 and U3 primers are located downstream of the Ubx promoter. (B, C) ChIP-qPCR analysis of anti-Asx and control rabbit IgG antibodies from wild-type embryos. The DNA recovered from ChIP samples was analyzed by qPCR and is shown along the y-axis. The signals are represented as mean ± SEM with N = 3. [file 13072_2017_151_MOESM9_ESM.pdf]

**Figure S4**

**% of input**

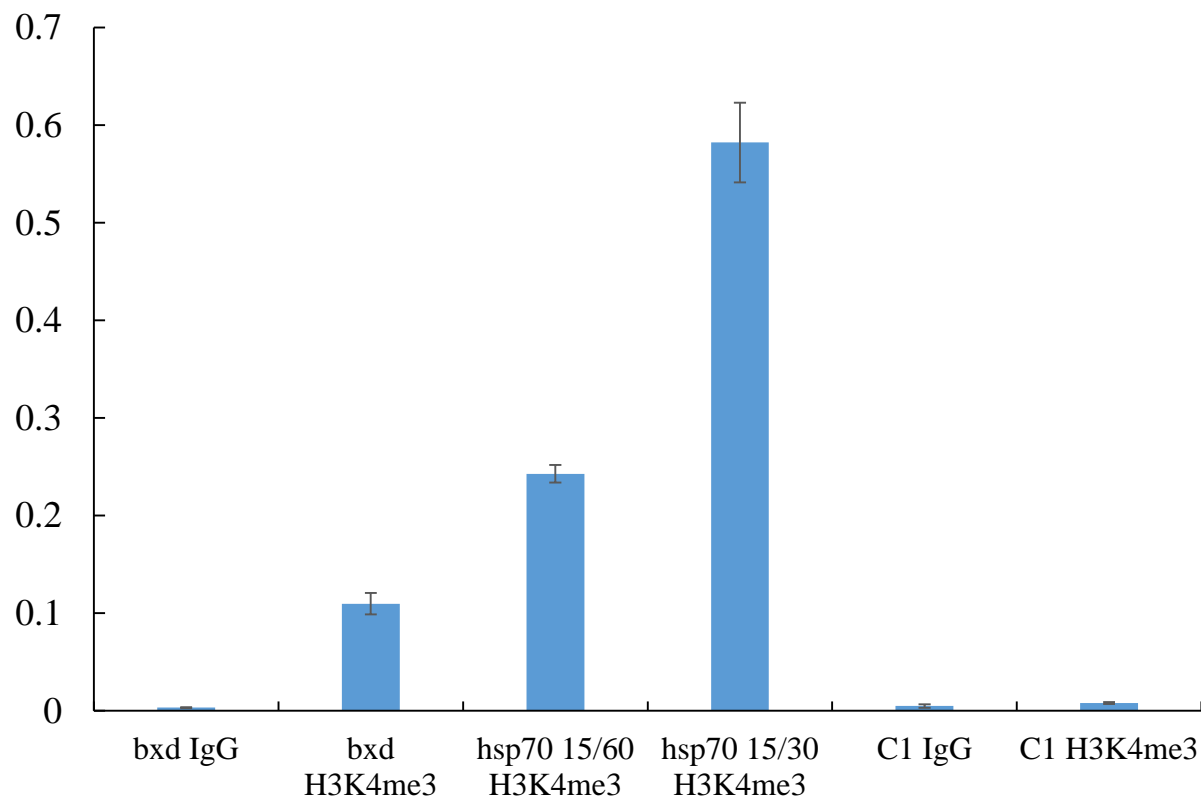

**H3K4me3**

**% of input**

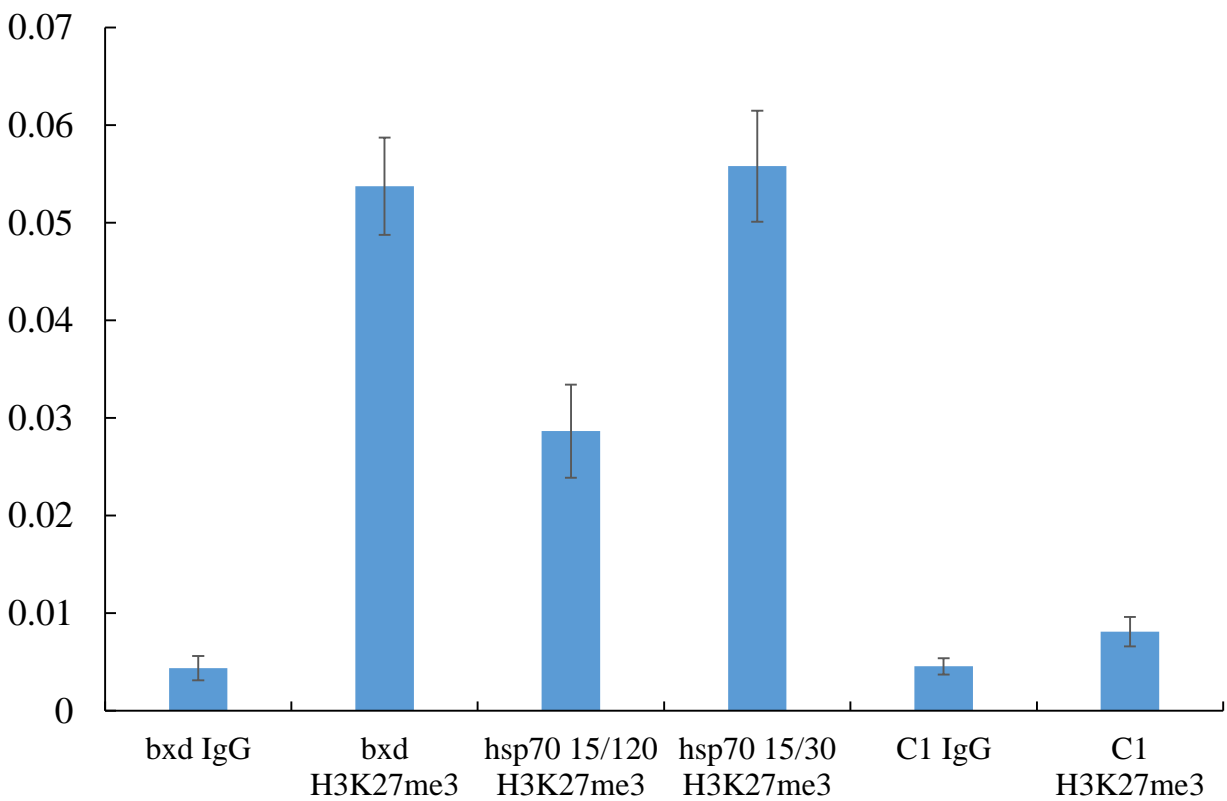

**H3K27me3**

Supplement: Supplementary file 10 — Additional file 10: Fig. S4. H3K4me3 and H3K27me3 levels at bithoraxoid (bxd) Polycomb response element (PRE) and DPR12 genes compared to highest and lowest levels observed at hsp70. ChIP-qPCR analysis of H3K4me3 and H3K27me3 and control rabbit IgG antibodies from wild-type embryos. The DNA recovered from ChIP samples was analyzed by qPCR, and percent recovery is shown along the y-axis. The data for hsp70 are taken from Fig 9. The signals are represented as mean ± SEM with N = 3. The bxd PRE primers are located between BX-C 218839 and 218959. C1 is located at +39kb to the DPR12 gene. [file 13072_2017_151_MOESM10_ESM.pdf]

**Figure S5**

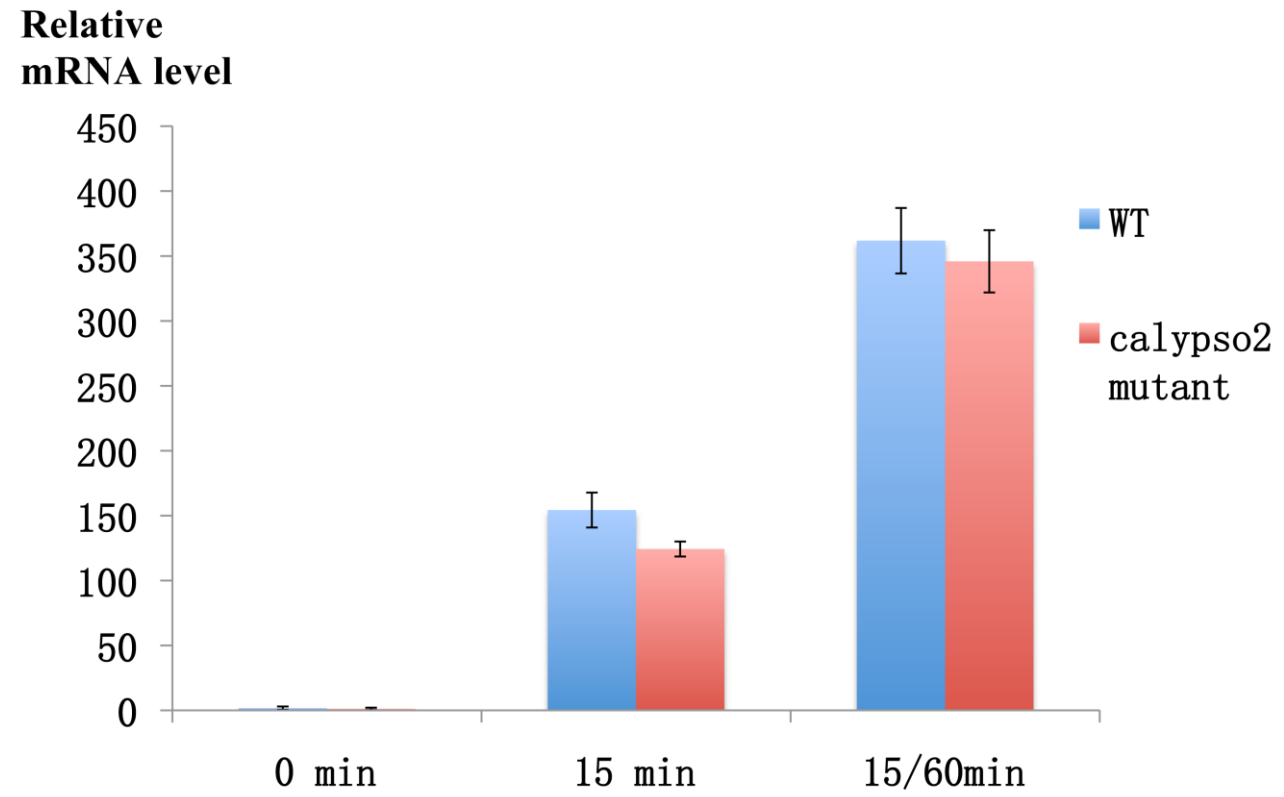

Supplement: Supplementary file 11 — Additional file 11: Fig. S5. calypso is not required for hsp70 repression during heat-shock induction and recovery. The relative mRNA levels in wild-type and calpyso 2 homozygous null mutant embryos were measured by RT-qPCR. The x-axis shows the heat-shock induction times at 37 °C, and heat-shock recovery times after 15 min of 37 °C heat-shock induction. The y-axis indicates the hsp70 mRNA level normalized to the control gene Ahcy89E mRNA level. The signals are represented as mean ± SEM with N = 3. [file 13072_2017_151_MOESM11_ESM.pdf]

**Figure S6**    % of input

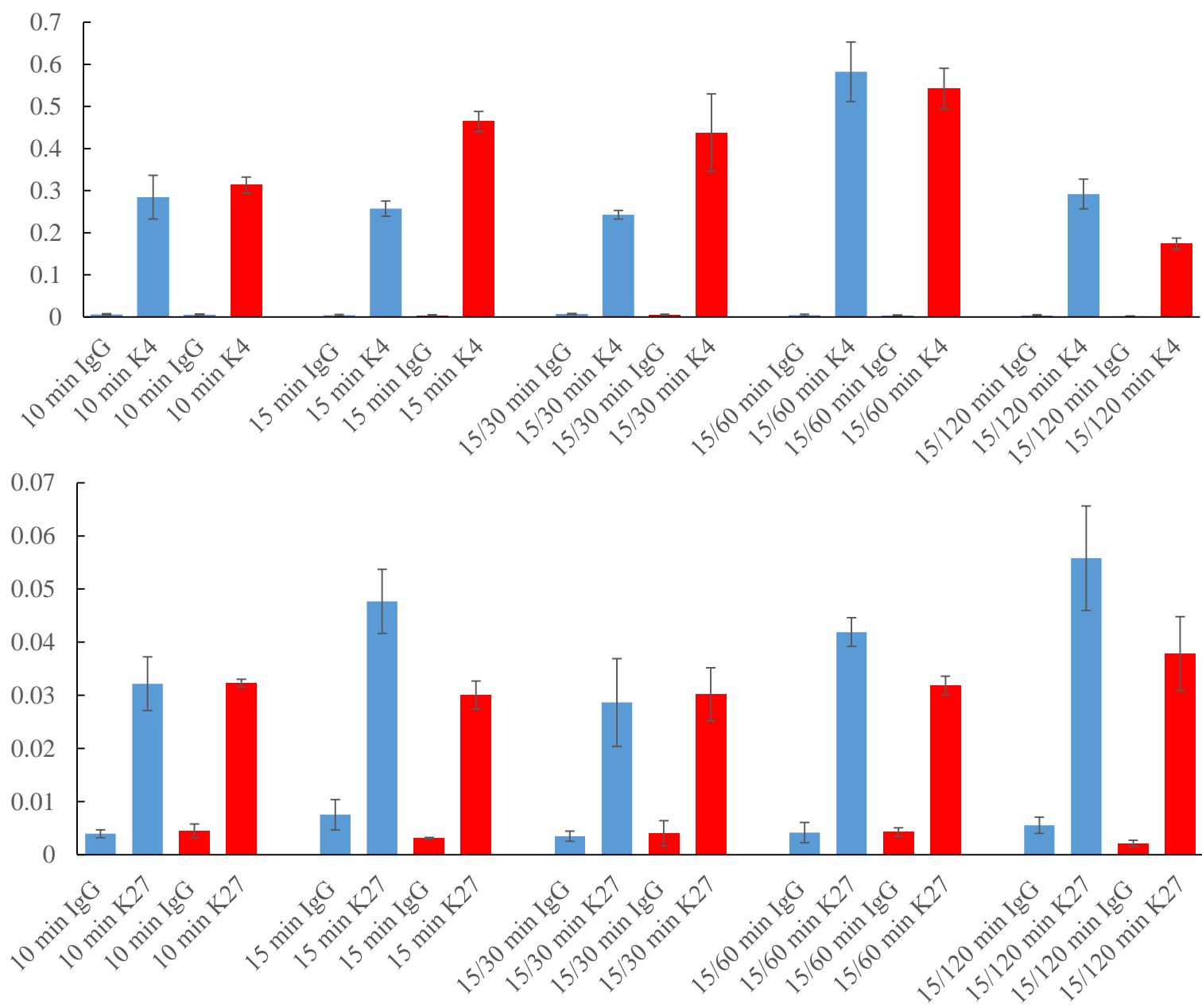

Supplement: Supplementary file 13 — Additional file 13: Fig. S6. Asx regulates H3K4me3 and H3K27me3 levels at hsp70. Both panels show ChIP-qPCR analysis comparing control rabbit IgG to trimethylated histones in wild-type (blue) and Asx 3 (red) embryos at different times of heat-shock induction and recovery as indicated in the x-axis. The y-axis indicates recovery after ChIP as a percentage of input DNA. The notation for the duration of heat shock/recovery is described in Fig. 7. All data are represented as mean ± SD. There was minimal difference when compared with the error bars using SEM as the error source in Fig. 9. [file 13072_2017_151_MOESM13_ESM.pdf]
