# Supplementary material for: Additional sex combs interacts with enhancer of zeste and trithorax and modulates levels of trimethylation on histone H3K4 and H3K27 during transcription of hsp70
Source: Epigenetics Chromatin. 2017 Sep 19;10:43. doi: 10.1186/s13072-017-0151-3 (PMC5605996; doi:10.1186/s13072-017-0151-3)
Supplement: Supplementary file 3 — Additional file 3: Table S1. PCR primer pairs for construction of E(z) SET and Asx expression vectors. [file 13072_2017_151_MOESM3_ESM.docx]

**Table S1**

PCR Primer Pairs for construction of E(z) SET and Asx expression vectors

| Asx COOH terminal deletion Constructs; (aa residues) | PCR Primer Pairs^a^ |
| --- | --- |
| A(1 – 610) | Forward; 5’-ccggatgactagt**catatg** AAA ACC ATT ACG CCG-3’  Reverse; 5’-ccggatcgatatc TCA^b^ GGC TAT TTG TCC TGT CG-3’ |
| A(1 – 354) | Forward; 5’-ccggatgactagt**catatg** AA A ACC ATT ACG CCG-3’  Reverse; 5’-ccggatcgatatc TCA CTT CTG ATT CTT GCA ATC G-3’ |
| A(1 – 200) | Forward; 5’-ccggatgactagt**catatg** AAA ACC ATT ACG CCG-3’  Reverse; 5’-ccggatcgatatc TCA CGC TGC TGT CGT CAG CTT C-3’ |
| A(200 – 354) | Forward; 5’-ccggtta**catatg** GCG CAG ATT GAG C-3’  Reverse; 5’-ccggtatcccggg TCA CTT CTG ATT CTT GCA ATC-3’ |
|  |  |
| Asx NH2 terminal deletion Constructs |  |
| A(1200 – 1669) | Forward; 5’-ccggttg**catatg** CAG CAG TTA CCCAACGTG -3’  Reverse; 5’-ccggattgcccggg TCA TCT AAT CAC ACA GGC GA -3’ |
| A(1200 – 1501) | Forward; 5’-ccggtt**catatg** ATG CAG CAG TTA CCC AAC-3’  Reverse; 5’-ccggttacccggg TCA TGT GTC CAC CGA TGC TGG AC -3’ |
| A(1381 – 1501) | Forward; 5’-ccggtt**catatg** TAT GTC CAA CGG AGG CCG-3’  Reverse; 5’-ccggttacccggg TCA TGT GTC CAC CGA TGC TGG AC -3’ |
| A(1501 – 1669) | Forward; 5’-ccggttg**catatg** ACA ACG GCT GGC AGC GGC-3’  Reverse; 5’-ccggattgcccggg TCA TCT AAT CAC ACA GGC GA-3’ |
|  |  |
| SET Domain (aa residues) |  |
| E(z) (626 – 740) | Forward; 5’-ccggcta**gaattc** ACA AGC ACC TGC TCA TGG CT-3’  Reverse; 5’-ccggatcctcgag TCA CTG TAG TCA AAG AAT AGC T-3’ |
|  |  |
| AsxETSR2(aa residues) |  |
| Asx (1200 – 150l) | Forward; 5’-ccggtt**gaattc** ATG CAG CAG TTA CCC AAC -3’  Reverse; 5’-ccggctcgag TCA TGT GTC CAC CGA TGC TGG AC -3’ |

-a lower case letters indicate vector sequences

-b termination codon underlined in reverse primer
