# Supplementary material for: Additional sex combs interacts with enhancer of zeste and trithorax and modulates levels of trimethylation on histone H3K4 and H3K27 during transcription of hsp70
Source: Epigenetics Chromatin. 2017 Sep 19;10:43. doi: 10.1186/s13072-017-0151-3 (PMC5605996; doi:10.1186/s13072-017-0151-3)
Supplement: Supplementary file 4 — Additional file 4: Text S2. Buffers. [file 13072_2017_151_MOESM4_ESM.docx]

**Text** **S2** Buffers

TEEZMG - 0.5 M KCl, pH7.9 (50 mM Tris-HCl/1 mM EDTA/1 mM EGTA/2.5 mM MgCl_2_/0.1 mM ZnSO_4_/10 mM β-mercaptoethanol/ 0.1% Brij 36T/5% glycerol)

TEMZG - 0.3 M KCl, pH 7.9 (50 mM Tris-HCl/1 mM EDTA/2.5 mM MgCl_2_/0.1 mM ZnSO_4_/10 mM β-mercaptoethanol/5% glycerol)

PBSMG - 0.3 M NaCl, pH 7.2 (PBS/2.5 mM MgCl_2_/5% glycerol/1 mM phenylmethylsulfonyl fluoride,(PMSF))

TEMZ - 0.1 M KCl, pH 9.0 (50 mM Tris-HCl/1 mM EDTA/2 mM MgCl_2_/5 µM ZnSO_4_/0.5 mM PMSF)

Dyl-TEEMZG - 0.1 M KCl, pH 7.9 (25 mM Tris-HCl, pH7.9/1 mM EDTA/1 mM EGTA/2 mM MgCl_2_/50 µM ZnSO_4_/10 mM β-mercaptoethanol/15 % glycerol/0.5 mM PMSF).

TZS - 0.3 M NaCl, pH7.9 (50 mM Tris-HCl/5 mM ZnSO_4_/50 mM 2-mercaptoethanol/10% Sarkosyl/protease inhibitors)

TZD - 0.3 M NaCl, pH7.9 (50 mM Tris-HCl/5 mM ZnSO_4_/10 mM 2-mercaptoethanol/3% Triton X-100/30 mM CHAPS/2 mM benzamidine-HCl/2 mM Na metabisulphite/1 mM PMSF)

TZXSC - 0.3 M NaCl, pH 7.9 (50 mM Tris-HCl/5 mM ZnSO_4_/10 mM 2-mercaptoethanol/3% Triton X-100/2% Sarkosyl/30 mM CHAPS/2 mM benzamidine-HCl/2 mM Na metabisulphite/1 mM PMSF)

TZGXSC - 0.3 M KCl, pH 7.9 (25 mM Tris-HCl/5 mM ZnSO_4_/1 mM DTT/5% glycerol/2% Triton X-100/1 % Sarkosyl/20 mM CHAPS/1 mM PMSF)

TZXSGC - 0.1 M NaCl, pH 9.0 (50 mM Tris-HCl/5 mM ZnSO_4_/1 mM DTT/5% glycerol/1% Triton X-100/0.5% Sarkosyl/10 mM CHAPS/0.5 mM PMSF)

Dyl-TZXS - 0.1 M NaCl/15% glycerol (50 mM Tris-HCl/5 mM ZnSO_4_/10 mM 2-mercaptoethanol/1% Triton X-100/0.5 % Sarkosyl/0.5 mM PMSF).

IM-A, pH7.9 (20 mM Tris-HCl, pH 7.9/1 mM EDTA/2.5 mM MgCl_2_/1 mM DTT/0.1% Brij 36T/20% glycerol/0.1 M NaCl/2 mM benzamidine-HCl, pH8.0/0.5 mM PMSF)

IM-PC (25 mM Tris-HCl, pH7.9/0.1 M NaCl/5 µM ZnSO_4_/1 mM DTT/5% glycerol/1% Sarkosyl/2% Triton X-100/0.5 M CHAPS/1 mM PMSF)

2X PDB-P5 (40 mM Tris-HCl, pH7.9/1 mM EDTA/1% Triton X-100/1 mM DTT/5% glycerol/30 mM NaCl/2 mM benzamidine-HCl/1 mM Na metabisulphite/2.5 µg/ml leupeptin/2.5 µg/ml bestatin/1 mM PMSF)

WB-0.6 M NaCl, pH7.9 (25 mM Tris-HCl/2 mM EDTA/5 µM ZnSO_4_/1 mM DTT/0.5% Triton X-100/2 mM benzamidine-HCl/1 mM Na metabisulphite/1 mM PMSF)

TELG (10 mM Tris-HCl, pH 7.6/1 mM EDTA/3.125 mM MgCl_2_/20% glycerol/ .01% Brij 36T/0.05 mM zinc acetate/0.5 mM PMSF/2 mM benzamidine-HCl/10 mM sodium sulphite)

Protease inhibitors (2 mM benzamidine/2 mM Na metabisulphite/2.5 µg/ml Aprotinin/0.08% bacitracin/2.5 µg/ml bestatin/2.5 µg/ml leupeptin/1 mM PMSF)

Protease and phosphatase inhibitors (0.5 mM PMSF/5 µg/ml leupeptin/0.02% soya bean trysin inhibitor/10 µg/ml pepstatin/5 µg/ml bestatin/0.08% bacitracin/2 trypsin inhibitor units aprotinin/2 mM benzamindine-HCl/2 mM metabisulphite/0.5 mM KF/0.2 mM Sodium Molybdate)
