# Supplementary material for: Additional sex combs interacts with enhancer of zeste and trithorax and modulates levels of trimethylation on histone H3K4 and H3K27 during transcription of hsp70
Source: Epigenetics Chromatin. 2017 Sep 19;10:43. doi: 10.1186/s13072-017-0151-3 (PMC5605996; doi:10.1186/s13072-017-0151-3)
Supplement: Supplementary file 5 — Additional file 5: Text S3. GST pull-down western assays of embryo nuclear extract. [file 13072_2017_151_MOESM5_ESM.docx]

**Text** **S3** GST pull-down Western assays of embryo nuclear extract

To confirm the activity of GST-AsxETSI-2 *ex vivo*, a reciprocal GST pull-down western assay was developed using cation Bio-Rex 70 fractionated embryo nuclear extracts as a source of E(z), coupled with a western blot assay. Nuclear extracts were prepared from 30 g of 6 -18 hour AEL embryos using a modification of the protocol of (1). All buffers were supplemented with the protease and phosphatase inhibitors (Additional File 4: Text S2).

For co-fractionation experiments, nuclear extract (AS-2) in buffer HEMG0.1, pH7.6 was applied to a Bio-Rex 70 (BR70) column equilibrated with buffer HEMG0.1 (2). The column was washed with 2.5 column volumes (cv) of buffer HEMG0.1 at 2 cv/h, and eluted with step gradients of 2.5 cv of 0.1 M, 0.18 M, 0.30 M, 0.60 M and 0.85 M KCl in buffer HEMG. Each fraction was flash frozen in liquid nitrogen and stored at -80^o^C. Western blot analysis indicated the presence of E(z) in the BR70-0.1 M fraction; Asx in both BR70-0.6 M and BR70-0.85 M fractions; and Trx in the BR70-0.85 M fraction.

To test interactions between E(z) and Asx fragments, 2 mg of the BR70-0.1 M fraction was adjusted with an equal volume of buffer 2X PD-NE/S1A, pH7.9 and mixed in 500 µL tubes with 1 g GµST-AsxETSI-2 immobilized on 10 µL GSH-Agarose (as described above) for 2 hr at 4^o^C, in parallel with GST-Agarose. The protein bound agarose beads were washed once with 350 µL buffer WB-0.1 M NaCl, pH7.9; 3 times with buffer WB-0.3 M NaCl, pH7.9 and once with buffer WB-0.1 M NaCl, pH7.9. The agarose pellet was mixed with 15 µL 2X SDS sample buffer, resolved by SDS-PAGE and transferred on to a nitrocellulose membrane for western blot analysis. The membrane was probed with 1:200 dilution of rabbit anti-E(z) IgG (Santa Cruz Biotech, cat# sc-98265), washed 3 times, blocked with nonfat milk, pH 9.0 and incubated in 1: 10,000 dilution of Donkey anti-rabbit IRDye680RD (LI-COR Biosci)/TBS/5% nonfat milk, pH 9.0 for 1 hr at room temperature. The membrane was washed 4 times and developed on the Odyssey infrared scanner (LI-COR Biosci).
