# Supplementary material for: Additional sex combs interacts with enhancer of zeste and trithorax and modulates levels of trimethylation on histone H3K4 and H3K27 during transcription of hsp70
Source: Epigenetics Chromatin. 2017 Sep 19;10:43. doi: 10.1186/s13072-017-0151-3 (PMC5605996; doi:10.1186/s13072-017-0151-3)
Supplement: Supplementary file 6 — Additional file 6: Table S2. Oligonucleotides for in situ PLA in Drosophila S2 cells. [file 13072_2017_151_MOESM6_ESM.docx]

**Table S2**

Oligonucleotides for *in situ* PLA in *Drosophila* S2 cells

| Oligonucleotides for Secondary Antibody conjugation | Oligonucleotide sequences |
| --- | --- |
| MTPX-GR-α-Rat-52mer (250 nmol)^a^ | **5′-amino**-AAA AAA AAA ATA TGA CAG AAC **GGA CGA TCA TCC AGC ACT AGT** AGA CAC TCT T-3’ |
| MTPX-Red-PLA- α -Rbb-52 mer (250 nmol) | **5′-amino**-AAA AAA AAA ATA TGA CAG AAC ATA CGG TCT CGC AGA TCG CTT AGA CAC TCT T-3’ |
| MTPX-PLA- α -Shp-35 mer (250 nmol) | **5′-amino**-AAA AAA AAA AGA CGC TAA TAG TTA AGA CGC T TUUU (2’O-Methyl RNA for U) |
|  |  |
| Circularization PLA Oligos |  |
| MTPX-Connector-65 mer (250 nmol) | **5′-PH^b^**-CTA TTA GCG TCC AGT GAA TGC GAG TCC GTC TAA GAG AGT AGT ACA GCA GCC GTC AAG AGT GTC TA-3’ |
| MTPX-Sp(Splint)25 mer (100 nmol) | **5′-PH**-GTT CTG TCA TAT TTA AGC GTC TTA A-3’ |
| MTPX-GR-Sp20 mer (100 nmol) | **5′-PH**-CTA GTG CTG GAT GAT CGT CC-3’ |
| MTPX-Red-Sp20 mer (100 nmol) | **5′-PH**-AGC GAA CTG CGA GAC CGT AT-3’ |
|  |  |
| Detector PLA Oligos (20 mer) |  |
| MTPX-GR-PLA-Oligo  (100 nmol) | **5′-Alexa 488**-CTA GTG CTG GAT GAT CGT CC-3’ |
| MTPX-Red-PLA-Oligo  (100 nmol) | **5′-Alexa 594**-AGC GAT CTG CGA GAC CGT AT-3’ |

-a scale of oligo synthesis

-b PH ; phosphorylated
