# Supplementary material for: Additional sex combs interacts with enhancer of zeste and trithorax and modulates levels of trimethylation on histone H3K4 and H3K27 during transcription of hsp70
Source: Epigenetics Chromatin. 2017 Sep 19;10:43. doi: 10.1186/s13072-017-0151-3 (PMC5605996; doi:10.1186/s13072-017-0151-3)
Supplement: Supplementary file 7 — Additional file 7: Text S4. Primers for hsp70, Ahcy89E, bxd PRE and Ubx promoters. [file 13072_2017_151_MOESM7_ESM.docx]

**Text** **S4** Primers for *hsp70*, *Ahcy89E*, *bxd* PRE and *Ubx* promoters

*hsp70*: +218 to +392 (Forward; 5’-TCA ACA AGT CGT TAC CGA GG-3’ and Reverse; 5’-CGT TCC GAA TCT GTG AAA GC-3’);

*Ahcy89E* (Forward; 5’-TCT GAT GTG TCC ACC TGC TC-3’ and Reverse; 5’ -GCC TTC CAA ATG CAC TCT TC-3’).

*bxd* PRE and *Ubx* promoter: L2 (Forward; 5’- TAT ATC GGA TCG CTT ATG GC -3’ and Reverse; 5’- CGC ATG GGG GAA CGC CGA TTG -3’), L7 (Forward; 5’- TAT CGA ACG AAG GGT ACC A -3’ and Reverse; 5’- GTC CGC AAA ACT AGC AAG TGC -3’), L8 (Forward; 5’- CAT GTG GAT CGC CGG CGT G -3’ and Reverse; 5’- GAT CGG AAT CCT GCA GCC AG -3’), U2 (Forward; 5’- TGA GTC GGC AGA GCA AAG T -3’ and Reverse; 5’- AAT GCC GCT GAT AAT GTG GA -3’) and U3 (Forward; 5’- AGG AAC AGC ACA GAA AAG CGA -3’ and Reverse; 5’- TCA AAG TAC GAG TTC ATT GCG -3’). *bxd* PRE primers used in Additional File 10: Fig S5 (Forward; 5’- CAA TTT GTC ACC GCA AGG CCA CTA -3’ and Reverse; 5’- GTC GCT TGT TTG GAT AAT TAC TTG GA -3’)

Control primers: rp49 (Forward; 5’- AAC CTG CTT CAA GAT GAC CA -3’ and Reverse; 5’- TCG ATA CCC TTG GGC TTG -3’) and C1 (39 kb upstream of the *DRP12* gene, see Fig S4) (Forward; 5’- CCG AAC ATG AGA GAT GGA AAA-3’ and Reverse; 5’- AAA GTG CCG ACA ATG CAG TTA-3’)
