# Supplementary material for: Additional sex combs interacts with enhancer of zeste and trithorax and modulates levels of trimethylation on histone H3K4 and H3K27 during transcription of hsp70
Source: Epigenetics Chromatin. 2017 Sep 19;10:43. doi: 10.1186/s13072-017-0151-3 (PMC5605996; doi:10.1186/s13072-017-0151-3)
Supplement: Supplementary file 8 — Additional file 8: Fig. S2. Alignment of amino acid sequences of AsxETSI. (A) Clustal Omega alignment of AsxETSI-1 and AsxETSI-2 showing 14.97 % sequence identity. (B) Clustal Omega alignment of AsxETSI-2 and ASXL1 (943–1307) showing 15.1% sequence identity. [file 13072_2017_151_MOESM8_ESM.pdf]

Figure S2

A

```
ETSI-1      ---------mktitpdtttttssqh-qqllipqadqhhqpmlqqqsllaappptmimehv          50
ETSI-2      mqqlpnvltmkmtlppsgvpttiaqqrlqpkmptgkgrk------atsnrlppgavnlers          54
                     ***: *. . ** :*:  *  :* .. ::       :    ** :: :*:

ETSI-1      nlvdddekdplaleqlevspstkht--------hslrrhlpriivkpippekk-------          95
ETSI-2      y--------qicqaviqnspnrenlkaqlrppaailnqhqptttapapinpvtlnvstv          106
                      :.   :: **. :.           *.:* *   . * * :

ETSI-1      ---pmaps----eeaavstapapptrlicsrriqqqqqvkaaaaaaaaaaaaaaaaaaaa          148
ETSI-2      aatpmsnittatgsmaaavaaappqnvlkqeellvs------------------------          142
               **:       . *.:.* *** .:: ...:  .

ETSI-1      qaqatssypsaispgskagtsqastmrevlasipgfsvkprrr---snkklt--------          197
ETSI-2      gavgagalpaglppn-vmgvgrpgvykvigprmsgfprkkyvqrkpsptlirhvfspgp          201
             * .:.: *:.: *    *..: .. : :   : **  *   :   * ..*

ETSI-1      -----taaqi------eqtkdgkidletp-----dsilas-----------tnlrallnk          230
ETSI-2      ggatataqqlqmlqqhhqsttspvpvqnpqqpapeqlihqngngqyvlvhranvgaadnq          261
                 ** *:      .*:. . : ::.*     :.:: .           :*: *  *:

ETSI-1      -qtfsllpplyqynliqllpsvdreaseleqpsssasggspseairlsasclnneffara          289
ETSI-2      aprassappmhqnqfvtvqn-----------plhsin-g---------------------          288
                *  **::* ::: :             *  *  . *

ETSI-1      clewrerlsegeftpenqlklkteaereknkldpwklkhfepfwgeknsrgkdkdklesd          349
ETSI-2      ----------------------------------------ipmggrgrpasvdt------          302
                                                     *: *.  .   . *.

ETSI-1      cknqk                354
ETSI-2      -----                302
```

B

```
AsxETSI-2        ------mqqlpnvltmktlppsgvptti---aqqrlqpkmptgkgrkatsnrlppgavnl      51
ASXL1943_1307    ltaeegldpldsltslwtvpsrggsdsngsycqqvdieklkin----gdsealsphgest      56
                       :: * .: :: *:*  *   :    .**    *:       . *: * * . .

AsxETSI-2        ersyqicqaviqnspnrenlkaqlrppaailnqhqptttapapinpvtlnvst------      105
ASXL1943_1307    dtasdfeghltedsseadtreaavtkgssvdkdekp-nwnqsaplskvngdmrlvtrtdg      115
                 : : ::   : ::* : :. :* :   ::: ::.:* . .  **:. *. ::

AsxETSI-2        ------------------------------------------vaatpmsnittatgsmaa      123
ASXL1943_1307    mvapqswvsrvcavrqkipdslllasteyqpravclsmpgssveatnplvmqllqgslpl      175
                                                           * **    :    **:

AsxETSI-2        avaaappqn--vlkqeellvsgavgagalpaglppnvmgvgrp-gvykvigpr-msgfpr      179
ASXL1943_1307    ekvlppahddsmsespqvpltkdqshgslrmgs---lhglgknsgmvdgsspsslralke      232
                   .  * ::  : :. :: ::   . *:*  *    : *:*:  *: .  .*  : .: .

AsxETSI-2        kkyvqrkpsptlirhvfspgpggatataqqlqmlqqhhqsttspvpvqnpqqpapeqli      239
ASXL1943_1307    pllpdscetgtglarieatqapgapqkncкavpsfd-------slhpvtnpitss-rkle      284
                     :   : * * *   : .**.   ..: :  ::       *  ** **   : .:*

AsxETSI-2        hqngngqyvlv---hranvgaadnqaprassappmhqnqfvtvqnplh-singipmggr-      294
ASXL1943_1307    emdskeqfssfscedqkevrams-qdsnsnaapgkspgdlttsrtprfsspnvisfgpeq      343
                 . :.: *:  .   .: :* * . *  .:.:**     ::.* :.* . * * * :* .

AsxETSI-2        -grp----asvd---------   301
ASXL1943_1307    tgralgdqsnvtgqgkklfgs   364
                  **     :.*
```
