# Supplementary material for: Additional sex combs interacts with enhancer of zeste and trithorax and modulates levels of trimethylation on histone H3K4 and H3K27 during transcription of hsp70
Source: Epigenetics Chromatin. 2017 Sep 19;10:43. doi: 10.1186/s13072-017-0151-3 (PMC5605996; doi:10.1186/s13072-017-0151-3)
Supplement: Supplementary file 12 — Additional file 12: Table S3. Standard deviation (SD) table for ChIP experiments. [file 13072_2017_151_MOESM12_ESM.docx]

**Table S3** Standard deviation (SD) table for ChIP experiments

| H3K4me3 Ab | WT IgG | WT K4me3 | *Asx^3^* IgG | *Asx^3^* K4me3 |
| --- | --- | --- | --- | --- |
| 10 min Avg. | 0.0059 | 0.2846 | 0.0053 | 0.3132 |
| 10 min SD | 0.0015 | 0.0512 | 0.0015 | 0.0190 |
| 15 min Avg. | 0.0038 | 0.2573 | 0.0034 | 0.4644 |
| 15 min SD | 0.0021 | 0.0180 | 0.0015 | 0.0238 |
| 15/30 min Avg. | 0.0072 | 0.2428 | 0.0040 | 0.4379 |
| 15/30 min SD | 0.0019 | 0.0104 | 0.0024 | 0.0921 |
| 15/60 min Avg. | 0.0039 | 0.5823 | 0.0033 | 0.5426 |
| 15/60 min SD | 0.0004 | 0.0707 | 0.0013 | 0.0482 |
| 15/120 min Avg. | 0.0032 | 0.2918 | 0.0017 | 0.1739 |
| 15/120 min SD | 0.0019 | 0.0354 | 0.0006 | 0.0135 |
|  | | | | |
| H3K27me3 Ab | WT IgG | WT K27me3 | *Asx^3^* IgG | *Asx^3^* K27me3 |
| 10 min Avg. | 0.0040 | 0.0322 | 0.0045 | 0.0323 |
| 10 min SD | 0.0007 | 0.0051 | 0.0013 | 0.0007 |
| 15 min Avg. | 0.0076 | 0.0477 | 0.0031 | 0.0300 |
| 15 min SD | 0.0028 | 0.0060 | 0.0001 | 0.0026 |
| 15/30 min Avg. | 0.0035 | 0.0287 | 0.0040 | 0.0302 |
| 15/30 min SD | 0.0009 | 0.0083 | 0.0023 | 0.0050 |
| 15/60 min Avg. | 0.0042 | 0.0419 | 0.0043 | 0.0318 |
| 15/60 min SD | 0.0019 | 0.0027 | 0.0008 | 0.0018 |
| 15/120 min Avg. | 0.0055 | 0.0557 | 0.0021 | 0.0378 |
| 15/120 min SD | 0.0015 | 0.0098 | 0.0006 | 0.0069 |
